# Social exclusion of older persons: a scoping review and conceptual framework

**DOI:** 10.1007/s10433-016-0398-8

**Published:** 2016-10-11

**Authors:** Kieran Walsh, Thomas Scharf, Norah Keating

**Affiliations:** 1 Irish Centre for Social Gerontology, National University of Ireland Galway, Galway, Ireland; 2 Institute of Health & Society, and Newcastle University Institute for Ageing, Newcastle University, Newcastle, UK; 3 Centre for Innovative Ageing, Swansea University, Swansea, UK; 4 Africa Unit for Transdisciplinary Health Research (AUTHeR), North-West University, Potchefstroom, South Africa

**Keywords:** Multidimensional disadvantage, Later life, Knowledge synthesis, Old-age exclusion

## Abstract

As a concept, social exclusion has considerable potential to explain and respond to disadvantage in later life. However, in the context of ageing populations, the construct remains ambiguous. A disjointed evidence-base, spread across disparate disciplines, compounds the challenge of developing a coherent understanding of exclusion in older age. This article addresses this research deficit by presenting the findings of a two-stage scoping review encompassing seven separate reviews of the international literature pertaining to old-age social exclusion. Stage one involved a review of conceptual frameworks on old-age exclusion, identifying conceptual understandings and key domains of later-life exclusion. Stage two involved scoping reviews on each domain (six in all). Stage one identified six conceptual frameworks on old-age exclusion and six common domains across these frameworks: neighbourhood and community; services, amenities and mobility; social relations; material and financial resources; socio-cultural aspects; and civic participation. International literature concentrated on the first four domains, but indicated a general lack of research knowledge and of theoretical development. Drawing on all seven scoping reviews and a knowledge synthesis, the article presents a new definition and conceptual framework relating to old-age exclusion.

**Electronic supplementary material:**

The online version of this article (doi:10.1007/s10433-016-0398-8) contains supplementary material, which is available to authorized users.

## Introduction

‘Social exclusion’ refers to the separation of individuals and groups from mainstream society (Commins 2004; Moffatt and Glasgow 2009). Widely applied in research, policy and practice spheres throughout Europe, the construct is also increasingly prevalent within political and scientific discourses in other world regions (Lee et al. 2014; Parmar et al. 2014). Building on a longstanding focus in European research on issues concerning disadvantage in later life (e.g. Townsend 1979), social exclusion is receiving growing attention within gerontology. Such interest reflects the combination of demographic ageing patterns, ongoing economic instability, and the susceptibility of ageing cohorts to increasing inequalities (Warburton et al. 2013; Bonfatti et al. 2015; Börsch-Supan et al. 2015; Scharf 2015). Older people who experience social exclusion tend to do so for a longer part of the life course than people belonging to other age groups (Scharf and Keating 2012).

While these features justify a scientific focus on old-age exclusion, research in the field remains under-developed. Critical and analytical perspectives on social exclusion are often absent from the international literature, and associated policy and practice responses (Levitas 1998; Scharf 2015). Social exclusion remains a highly contested concept with definitions frequently lacking agreement and transferability (Silver 1994; Morgan et al. 2007; Abrams and Christian 2007; Börsch-Supan et al. 2015) and criticised for homogenising experiences of exclusion (Levitas 1999, 2006). Consequently, social exclusion is prone to considerable ambiguity (Bradshaw 2004). This is unsurprising given the context-specific nature of exclusion, and its objective and subjective effects on individuals, groups and societies (Room 1999; Chamberlayne et al. 2002).

Issues of ambiguity are especially evident for old-age exclusion (Scharf and Keating 2012). This occurs for two reasons. First, while older people are identified as a group facing heightened risks of exclusion, little is known about the ways in which ageing and exclusion intersect across the life course (Scharf et al. 2005; Börsch-Supan et al. 2015). Instead, research disproportionately focuses on labour market integration (Madanipour 2011) and on the exclusion of people of working age, those with low-incomes, and children and youth (Moffatt and Glasgow 2009). Such a focus often overlooks the position of older people, with a general lack of research on social exclusion and ageing. There is also a paucity of research on ageing individuals of different social locations (e.g. gender, ethnicity, disability). Second, knowledge deficits can be attributed to disjointed evidence concerning older-adult disadvantage. Research is spread across the sub-fields of gerontology and related disciplinary fields rather than being drawn together in a single coherent discourse on exclusion. Notwithstanding recent contributions (e.g. Scharf and Keating 2012; Warburton et al. 2013; Börsch-Supan et al. 2015), few attempts have been made to review existing evidence pertaining to old-age exclusion. The lack of knowledge synthesis not only limits what can be said about ageing and exclusion in empirical terms, but also inhibits the development of critical understandings of exclusion within gerontology. Further, it restricts the formulation of meaningful conceptualisations concerning potential linkages between processes of exclusion and the wellbeing of ageing adults.

Nevertheless, social exclusion can offer valuable insight into the complexity of disadvantage affecting older individuals and groups (Room 1995, 1999; Béland 2007; Scharf 2015). Its capacity to account for both relational and distributional forms of disadvantage offers a comprehensiveness typically ignored in other conceptions (Gough et al. 2006). There is even perceived value in its ambiguity, given that this enhances the flexibility of the concept to reflect different contexts, thereby increasing its conceptual power (Levitas 1998; Abrams and Christian 2007). Therefore, if appropriately interrogated and tested within gerontology, social exclusion could be helpful in deconstructing multidimensional disadvantage in later life (Myck et al. 2015). It offers the potential to understand life-course features of old-age disadvantage, including cumulative inequalities and the changes that occur in exclusionary mechanisms over time. Crucially, social exclusion can also illuminate individual, structural and societal components of marginalisation (Saunders 2008), including such social categorisations and locations as gender, social class, ethnicity and sexual orientation. Thus, unlike allied concepts of poverty and deprivation, it provides a means to understand the dynamic and multi-level construction of old-age disadvantage (Room 1995, 1999). Given the growing influence of demographic ageing on European and international policy agendas, typically reflecting a burden-discourse (Phillipson 2013), a specific focus on old-age exclusion may offer a valuable approach for informing and evaluating age-related social policy. It is also likely to be particularly relevant given prevailing economic austerity in Europe and elsewhere, and the potential of austerity to reduce older people’s inclusion (Walsh et al. 2015).

Due to the lack of knowledge synthesis and the potential value of an exclusionary perspective, this article seeks to advance debates on old-age exclusion. Drawing on the findings of seven scoping reviews, the article synthesises knowledge on social exclusion of older persons, and proposes a conceptual framework of the phenomenon.

## Defining social exclusion of older persons

Defining social exclusion is often a function of disciplinary perspectives, context and even political efforts to address disadvantage (Silver 1995; Morgan et al. 2007). The conceptual evolution of social exclusion can be traced to a number of theoretical traditions (Silver 1994), the first of which relates to the semantic origins of the concept within French sociology. This perspective emphasises the dynamic and processual nature of exclusion across relational, symbolic and economic dimensions (De Haan 1998). A key tenet here relates to French Republican rhetoric around the moral integration discourse of ‘solidarity’, the ‘social contract’ (Silver 1995). Concern is expressed for the weakening or rupture of the social bond, which introduces risks for the individual in terms of “material and symbolic exchange with the larger society” (Silver 1995). Silver (1994) refers to this tradition in its contemporary form as the *solidarity paradigm*.

By contrast, the social exclusion concept in the Anglo-Saxon tradition emerged from critical social policy and debates about disadvantage. Townsend’s work on reconfiguring perspectives of poverty influenced the establishment of a more comprehensive discourse on disadvantage that was underlined by social democratic principles and ideas of the ‘underclass’ (Silver 1994). The Anglo-Saxon perspective emphasised a move towards considering citizenship rights, the ability to participate fully in society, and the power imbalance emanating from coercive hierarchal societal structures (Silver 1994; De Haan 1998). Silver (1994) terms this tradition the *monopoly paradigm*.

Silver (1994) also refers to a third model, the *specialization paradigm*, which was influential in US and UK discourses. In this paradigm, liberal ideologies underline notions of contractual and voluntary exchanges of rights and obligations, where individual differences give rise to specialisation in competing spheres involving the market and social groups. Exclusion is seen as a product of discrimination, the liberal state’s lack of enforcement or inappropriate enforcement of rights, barriers to movement/exchange between spheres, and market failures (De Haan 1998).

Definitions of social exclusion reflect these different theoretical traditions and vary in their emphasis on the constructs of solidarity and power. However, overlap across these elements has been identified within international research and policy perspectives (De Haan 1998). No definitions focus heavily on gender, social class, ethnicity or sexuality. That said, several definitions specify how exclusion affects individuals and groups.

Regardless of its differing origins, social exclusion is characterised by at least four common features. Firstly, it is a *relative* concept (Atkinson 1998). Scharf and Keating (2012) highlight the centrality of identifying which population base older-adult exclusion should be assessed against. For example, should the ‘normative’ integration levels experienced by the general population be used or those experienced by the older population? Secondly, exclusion involves *agency*, where an act of exclusion is implied (Atkinson 1998). This might involve older individuals being excluded against their will, lacking the agency to achieve integration for themselves, or choosing to exclude themselves from mainstream society. Thirdly, exclusion is *dynamic* or *processual*, with individuals and groups moving in and out of exclusion and experiencing different forms of exclusion over time. (Scharf 2015). Fourthly, most definitions acknowledge the multidimensionality of exclusion (Béland 2007; Billette and Lavoie 2010; Levitas et al. 2007; Scharf and Keating 2012). For example, Walker and Walker (1997) refer to *social, economic, political or cultural systems*. Multidimensionality is particularly important for older people given that research on social exclusion and ageing highlights the impact of exclusion on various life domains (e.g. Grenier and Guberman 2009; Walsh et al. 2012a; Hrast et al. 2013).

Many existing definitions (see supplementary material for presentation of reviewed definitions) reflect features of relativity, agency, dynamism and multidimensionality. To assist in setting the parameters of our scoping reviews, we draw on Levitas et al. (2007) to construct a working definition that acknowledges the potential of demographic ageing to intersect with exclusionary processes. It states that:

Social exclusion of older persons is a complex process that involves the lack or denial of resources, rights, goods and services as people age, and the inability to participate in the normal relationships and activities, available to the majority of people across the varied and multiple domains of society. It affects both the quality of life of older individuals and the equity and cohesion of an ageing society as a whole (Adapted from Levitas et al. 2007).

## Methodology

### Study design and research questions

A two-stage methodology, involving seven individual scoping reviews, was undertaken. A scoping review is a means of summarising current research knowledge and identifying gaps in existing research (Arksey and O’Malley 2005; Grant and Booth 2009). Our approach drew on the framework developed by Arksey and O’Malley (2005) and expanded by Levac et al. (2010). It involved: (1) identifying the research question; (2) identifying relevant studies; (3) study selection; (4) charting the data; and (5) collating, summarising and reporting the results.

The full body of literature pertaining to old-age exclusion may be conceptual and empirical; scattered across different literatures; specific to only one exclusion domain (e.g. financial and material resources); and may not even be labelled or referred to as exclusion. Additionally, within specific domains, there might be several dimensions and distinct literatures (e.g. poverty in the financial and material resources domain). To address this challenge a two-stage scoping review, with targeted but interconnected research questions, was developed.

In stage one, the focus was on frameworks presenting full conceptualisations of old-age exclusion. Frameworks had to involve a detailed articulation of how social exclusion can occur in older people’s lives, and particularly the multiple domains of exclusion. The research question guiding this stage was as follows: *How is social exclusion of older people conceptually constructed?*

Findings from stage one directly informed stage two. Here, the focus was on reviewing empirical and conceptual literature on each domain of social exclusion identified in the review of conceptual frameworks. Thus, the research question for stage two was as follows: *What are the main themes, or dimensions, documented in the international literature in relation to each domain?* The domains included in stage two are outlined when presenting findings from the first stage. Across the two-stage process, seven individual scoping reviews were conducted. One review addressed conceptual understandings of old-age exclusion, and six further reviews focused on each domain, respectively. All scoping reviews were completed by November 2015.

### Study selection, inclusion/exclusion criteria and screening material

Study selection followed a team approach (Levac et al. 2010). Inclusion/exclusion criteria, data sources and search terms were agreed and refined by the authors, with decisions to exclude or include ambiguous texts confirmed by two or more team members. The inclusion criteria were as follows: (1) gerontological literature since 1997; (2) academic, peer-reviewed journal articles, books, and research reports that present original conceptual/empirical work; and (3) documents with a focus on older people (aged 50 years and over). Excluded from the review were dissertations, theses and conference papers, EU and national policy documents, texts referring only to ‘social inclusion’,^1^ other scoping reviews, and documents published in languages other than English. For stage one, we included documents that present a conceptual framework of old-age exclusion. For stage two, we included documents that present information relating to exclusion of older people in a particular domain.

Search keywords were derived from the established literature on old-age exclusion. Keywords relating to exclusion included: social exclusion; disadvantage; vulnerability; risk; cumulative disadvantage. Keywords relating to ageing and older people included: ag(e)ing; older persons; older adults; seniors; elderly; elders; senior citizens. Keywords specific to stage one included framework, model, conceptual model/framework and theoretical frame. Stage-two domain-specific keywords were generated after domains were identified in stage one and are presented with the stage-two findings.

A diverse set of electronic bibliographic databases were chosen to maximise the comprehensiveness of the review: AgeLine (EBSCO); Applied Social Sciences Index and Abstracts; ScienceDirect; Scopus; Web of Science; and PsycINFO. Google Scholar and Google Books were also searched. The first 1000 articles of search returns were considered, or until lack of relevance was established. The decision to include or exclude articles began with a title review, followed by abstracts of papers, executive summaries of reports, and introductions of books examined for relevance. The full text of eligible papers was then reviewed. After completing this step, texts that still fulfilled the inclusion criteria were included in the final sample. The bibliographic management system EndNote was used to track the documents included in each review step. Overall, 444 documents across stages one and two were included in our analysis.

### Data charting, analysis and reporting

Key information was extracted from each document in the final sample and charted using a descriptive analytical method and Microsoft Excel data-charting forms (Arksey and O’Malley 2005). In addition to bibliographic details, the forms collected information on study methodology (design/approach, sample, data collection technique) and the structure of the conceptual frameworks (stage one) and empirical/conceptual findings (stage two). As suggested by Levac et al. (2010), a qualitative content analysis was then performed on the information collected in the forms.

## Stage one findings: conceptual frameworks of social exclusion of older persons

Eight documents presented conceptual frameworks on old-age exclusion (see supplementary material for flow diagram of stage one), highlighting a limited relevant literature. However, two other bodies of work, encompassing 17 texts, were relevant to the stage one question, and this material will be outlined first. Twelve of these documents discussed the conceptualisation and theorisation of the multidimensional nature of age-related exclusion. Scharf and Keating’s (2012) edited book interrogates traditional understandings through an ageing lens. Börsch-Supan et al. (2015), presenting data from the Survey of Health, Ageing and Retirement in Europe (SHARE), explore the social, economic and individual components of exclusion. Warburton et al. (2013) chart a theoretical analysis of the social inclusion/exclusion of older people. Lui et al. (2011) identify economic deprivation, cumulative disadvantages, social participation and civic engagement, and cultural recognition as key challenges, in their critique of the Australian social inclusion approach. Scharf et al. (2001) refer to participation and integration, spatial segregation, and institutional disengagement as key exclusion themes, while Scharf (2015) examines the role of economic austerity in constructing and exacerbating old-age exclusion. The other texts are more operational in nature, emphasising the multidimensionality of exclusion and its risk factors (Patsios 2000; Ogg 2005; Hoff 2008; Hrast et al. 2013; Lee et al. 2014; Myck et al. 2015).

The remaining five texts refer to complex age-related disadvantage constructs. These are akin to old-age exclusion, but do not reference social exclusion. They highlight many of the components inherent within our old-age exclusion working definition. Three articles address life-course factors and their relationship to age-related inequalities. Dannefer (2003) reflects on how social processes may interact to produce stratification and differential distribution of opportunities in later life. Dewilde (2003) develops an analytical life-course framework for exploring exclusion and poverty, emphasising the influence of life-course experiences and status positions within different domains. Cavalli and Bickel (2007) outline how critical life-events can exacerbate the potential for old-age relational exclusion. Two texts deal with notions of vulnerability. Grundy (2006) conceptualises vulnerability of older people as an imbalance between challenges and a set of reserve capacities (e.g. financial resources; family and social support), while Schröder-Butterfill and Marianti’s (2006) framework is structured around exposure, threats, coping capacities and outcomes.

The eight documents presenting conceptual frameworks offer original conceptual frameworks on social exclusion of older persons (Guberman and Lavoie 2004; Scharf et al. 2005; Barnes et al. 2006; Jehoel-Gijsbers and Vrooman 2008; Feng 2003; Walsh et al. 2012a) and extended versions of these original conceptualisations. Scharf and Bartlam (2008) extended the work by Scharf et al. (2005), and Kneale (2012) extended the work of Barnes et al. (2006). These eight documents represented the final sample for stage one.

The basis for and level of conceptualisation varies across frameworks. Guberman and Lavoie (2004) developed their framework from a set of thematic areas identified within the international literature. While Scharf et al. (2005) and Barnes et al. (2006) also draw on the literature, their frameworks are used to inform an operational assessment of old-age exclusion. The former focuses on a survey of 600 older adults in socially deprived neighbourhoods. The latter draws on the wave 1 sample of the English Longitudinal Study of Ageing (ELSA). Conversely, Feng’s (2003) framework is solely empirical and based on analysis of six surveys conducted across China. Walsh et al. (2012a) use a combined approach, deriving a working model of age-related rural exclusion from the existing literature, and then refining this on the basis of 106 qualitative interviews with rural-dwelling older people. The frameworks of Jehoel-Gijsbers and Vrooman (2008), Scharf and Bartlam (2008), and Kneale (2012) build on previous conceptualisations. Jehoel-Gijsbers and Vrooman (2008) refined and adapted an earlier conceptualisation of social exclusion (Jehoel-Gijsbers and Vrooman 2007) from the general population to older adults, analysing data from the European Social Survey (2002), the EU Statistics on Income and Living Conditions survey (2005), and the 2004 wave of SHARE. Scharf and Bartlam (2008) extended the framework of Scharf et al. (2005) to rural contexts. Kneale (2012) built on Barnes et al. (2006) to analyse wave 4 of ELSA. Accordingly, the frameworks of Scharf and Bartlam (2008) and Scharf et al. (2005), and of Kneale (2012) and Barnes et al. (2006) are grouped together in this article.

With reference to Table 1, each framework embraces a full model of participation, articulating a set of domains across which older people can experience exclusion. The old-age exclusion presented in these conceptualisations is multidimensional. In five conceptualisations, the domains represent both processes and outcomes of exclusion (Guberman and Lavoie 2004; Jehoel-Gijsbers and Vrooman 2008; Scharf et al. 2005; Scharf and Bartlam 2008; Barnes et al. 2006; Kneale 2012; Walsh et al. 2012a). Several frameworks also point to interconnections between domains (Guberman and Lavoie 2004; Scharf et al. 2005; Scharf and Bartlam 2008; Barnes et al. 2006; Kneale 2012; Walsh et al. 2012a), with a lack of financial resources, for instance, impinging on access to services. These characteristics are used by some authors (Guberman and Lavoie 2004; Jehoel-Gijsbers and Vrooman 2008; Scharf et al. 2005; Scharf and Bartlam 2008; Walsh et al. 2012a) to emphasise the dynamic nature of exclusion. In such formulations, old-age exclusion can change in form and degree of impact over the course of later life. Frameworks supported by quantitative data analysis, such as Scharf et al. (2005); Scharf and Bartlam (2008) and Barnes et al. (2006); Kneale (2012), point to older people simultaneously experiencing more than one domain of exclusion.

**Table 1:** Conceptual frameworks of social exclusion of older persons

Summary exclusion domains	Guberman and Lavoie (2004)	Scharf et al. (2005); Scharf and Bartlam (2008)	Barnes et al. (2006); Kneale (2012)	Jehoel-Gijsbers and Vrooman (2008)	Feng (2003)	Walsh et al. (2012a)
Material and financial resources	1. Economic exclusion	1. Exclusion from material resources	1. Exclusion from material resources/common consumer goods 2. Exclusion from financial products	1. Socio-economic exclusion: material deprivation	1. Economic situation	1. Income and financial resources
Services, amenities and mobility	2. Institutional exclusion (e.g. decreased services)	2. Exclusion from basic services	3. Exclusion from basic services 4. Local amenities	2. Socio-economic exclusion: social rights (e.g. exclusion from government provisions)	2. Social rights	2. Access to services 3. Transport and mobility
Social relations	3. Exclusion from meaningful relations	3. Exclusion from social relations	5. Exclusion from social relationships	3. Socio-cultural exclusion: social integration (e.g. lack of social relations)	3. Social participation 4. Perceptions of loneliness 5. Social support 6. Social integration	4. Social connections and social resources
Civic participation	4. Socio-political exclusion	4. Exclusion from civic activities	6. Exclusion from civic activities and access to information			
Neighbourhood and community	5. Territorial exclusion	5. Neighbourhood exclusion	7. Neighbourhood exclusion			5. Safety, security and crime
Socio-cultural aspects of society	6. Symbolic exclusion (e.g. negative representations of certain groups) 7. Identity exclusion (e.g. reduction to single identity such as age)		8. Exclusion from cultural activities	4. Socio-cultural exclusion: normative integration (e.g. lack of integration with society’s norms and values)		

Only half of the frameworks explicitly acknowledge agency in the exclusion of older people, with the others implying its role. Society through its practises, norms and bureaucracies, and individuals through their limited capacities, choices and adoption of societal norms produce exclusion. Guberman and Lavoie (2004) go further by highlighting how socio-political exclusion relates to a lack of individual power and agency. Walsh et al. (2012a) note that personal agency, and a sense of independence, can mediate exclusionary experiences. Jehoel-Gijsbers and Vrooman (2008) offer the most detailed analysis, acknowledging the agency of multiple actors, including individuals, communities, organisations, and governments, in creating and/or protecting against exclusion. The relative nature of exclusion is primarily implied, with frameworks grounded in a specific jurisdiction or place-based setting (e.g. rural Ireland/Northern Ireland in Walsh et al. 2012a). Barnes et al. (2006); Kneale (2012) offer the exception, with old-age exclusion set relative to the welfare of the general older population.

Theoretical traditions of social exclusion are evident in several conceptual frameworks. For instance, Scharf et al. (2005), Scharf and Bartlam (2008) broadly reflect the Anglo-Saxon tradition, while Guberman and Lavoie’s (2004) focus on symbolic and identity exclusion aligns with French sociological understandings. However, this categorisation risks an oversimplification, with several frameworks incorporating aspects of both traditions. Jehoel-Gijsbers and Vrooman (2008) acknowledge a combination of structural features of the Anglo-Saxon tradition and socio-cultural elements of the French-Republican tradition. Of greater relevance to old-age exclusion is arguably the influence of critical gerontology perspectives in three frameworks (Guberman and Lavoie 2004; Scharf et al. 2005; Scharf and Bartlam 2008; Walsh et al. 2012a).

A number of frameworks offer insight into the causalities of old-age exclusion. Operationally orientated conceptualisations (e.g. Barnes et al. 2006; Kneale 2012) highlight particular risk associations (e.g. living alone; gender; ethnicity; age 85 years plus). It is primarily in this manner that frameworks deal with social categorisations, such as gender, social class, and ethnicity, but with variations in the direction of associations across different domains. For example, Kneale (2012) found that while gender was not a significant predictor of overall exclusion, it was connected to certain individual domains (e.g. older women were more likely to be excluded from cultural activities, and less likely to be excluded from social relationships); Jehoel-Gijsbers and Vrooman (2008) showed that education-level, lower income and poorer health were more likely to mean older adults were in the most excluded group; Scharf et al. 2005 showed that older people belonging to particular ethnic minority communities (i.e. Pakistani and Somali older people) were more likely to be excluded from material resources, social relations and basic services. Sexual orientation was the notable exception from all frameworks. Within several frameworks, authors note that assessing the relationship between social categorisations and exclusion is problematic given their correlation with other risk factors, such as living alone and income (Barnes et al. 2006).

Elaborating in more conceptual depth on potential drivers of old-age exclusion, Jehoel-Gijsbers and Vrooman (2008) take an expanded view of risk factors. The authors highlight the influence of macro risks surrounding social processes (e.g. population ageing; economic recession; individualisation) and government policy/provision (e.g. inadequate policy and provision), meso risks relating to official bodies, business and citizens (e.g. discrimination; inadequate implementation), and micro risks at the individual/household level (e.g. health, labour market position). Walsh et al. (2012a) describe the influence of individual capacities (e.g. personal agency; adaptive capacity; risk management), life-course trajectories (e.g. transitions around bereavement; health and dependency; ageing), place characteristics (e.g. natural elements; community cohesion; attachment and belonging), and macro-economic forces (e.g. changing economic structure and service retrenchment; economic conditions and emigration) in mediating rural age-related exclusion. In their description of symbolic and identity exclusion, Guberman and Lavoie (2004) note the cultural and societal drivers of individual and group disenfranchisement.

In general, however, most old-age exclusion frameworks focus less on disentangling the complexity surrounding drivers of exclusion, than on articulating the various domains. Reflecting the empirical or operational nature of most frameworks, there is in fact a tendency to neglect a detailed theoretical explanation of why exclusion occurs in old age. This is in terms of: how macro, meso and micro factors combine and interact to construct or protect against multidimensional old-age exclusion; how ageing as a life-course process can increase susceptibility to multidimensional exclusion; and how outcomes in particular domains function as components in other forms of exclusionary processes to construct multidimensional old-age exclusion. Such a gap in conceptual understanding represents a significant limitation of many existing frameworks. The research, policy and practice challenge of multidimensional old-age exclusion must therefore be viewed in this context of somewhat stagnated conceptual development.

In summary, stage one findings illustrate the general lack of conceptualisation with respect to old-age exclusion. The findings, however, do illustrate cross-cutting themes evident across framework domains (Table 1). These can be broadly labelled as follows: material and financial resources; social relations; services, amenities and mobility; civic participation; neighbourhood and community; and socio-cultural aspects of society. We offer these six themes as the synthesised domains of exclusion established from state-of-the-art knowledge, and utilise them as a basis for stage two of the review.

## Stage two findings: domains of social exclusion of older persons

**Fig. 1:**
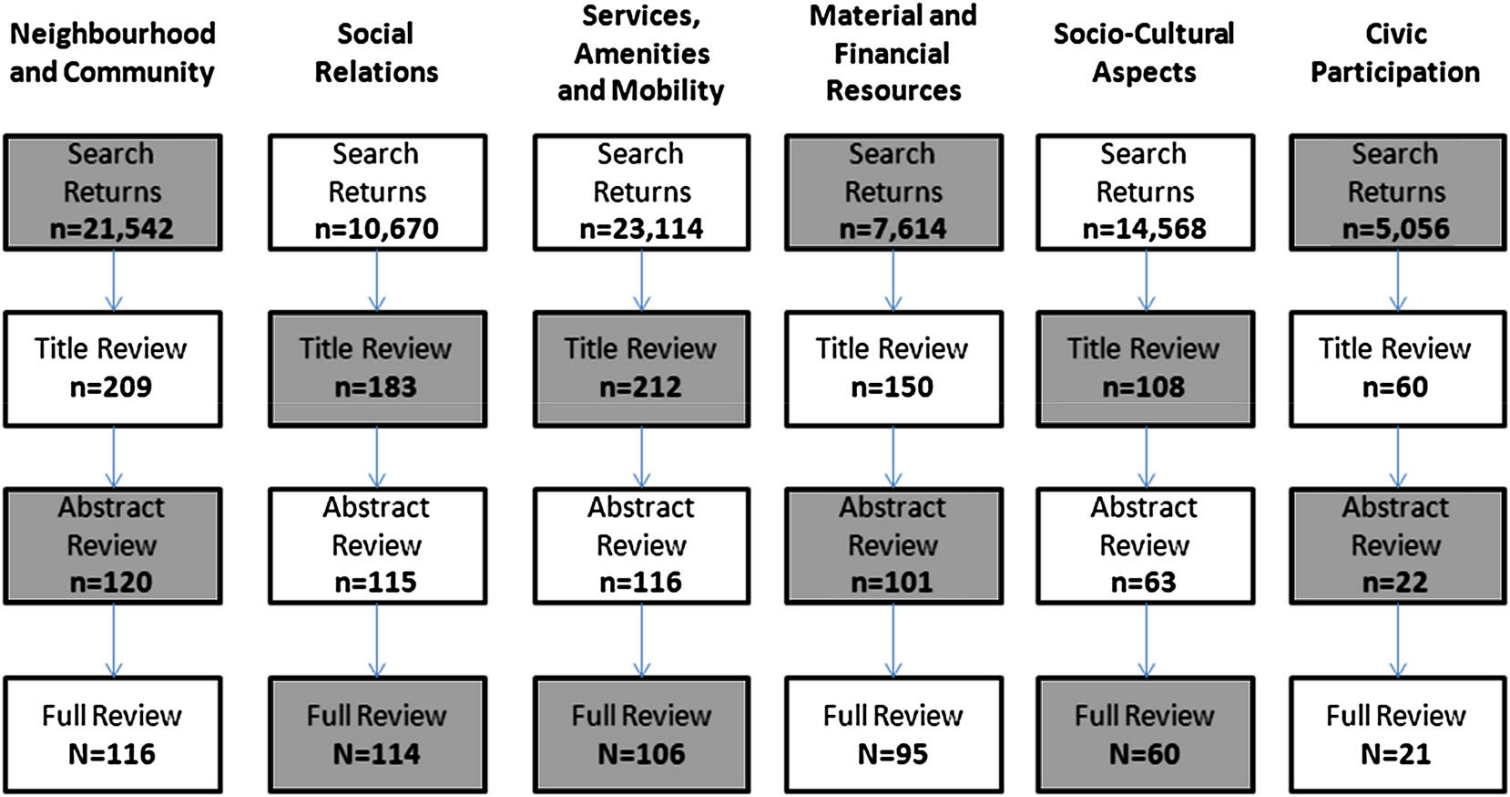
Stage two breakdown of review process

Scoping reviews were conducted for each of the six domains identified in stage one. Figure 1 summarises the number of texts included in each step of each review. It also illustrates the domains prioritised in published research (e.g. neighbourhood and community) and those that have received less attention (e.g. civic participation). Taking account of the overlap among identified documents across domains, 425 texts were identified in total, with some documents from stage one also included.

The scoping reviews for five domains identified what we term *context*-*oriented* texts, which consider domain topics together with multiple other factors, but do not feature extensive interpretation of domain-specific exclusionary relationships. As this body of work adds to the broad evidence base, it is acknowledged within each domain. However, these texts are not considered in detail since they contribute little to explicit understandings of exclusion in later life. Excluding these context-oriented papers, Table 2 presents a breakdown of key characteristics of the reviewed material for the domains, identifying trends with respect to sources, methodological approaches and common conceptual features of exclusion.

**Table 2:** Breakdown of key characteristics of domain-specific final review sample

	Neighbourhood and community	Social relations	Services, amenities and mobility	Material and financial resources	Socio-cultural aspects of society	Civic participation
National source (top 3)						
	UK (31 %)	UK (17 %)	UK (38 %)	UK (25 %)	UK (30 %)	UK (21 %)
	Australia (11 %)	US (13 %)	Australia (11 %)	US (17 %)	US (18 %)	5 equal sources
	US (9 %)	Australia (9 %)	Canada (10 %)	Australia (8 %)	Ireland (13 %)	
Methodology (top 3)						
	Qualitative (49 %)	Quantitative (57 %)	Quantitative (40 %)	Quantitative (61 %)	Descriptive analysis^a^ (50 %)	Descriptive analysis^a^ (37 %)
	Quantitative (16 %)	Qualitative (32 %)	Qualitative (28 %)	Descriptive analysis^a^ (15 %)	Qualitative (28 %)	Qualitative (37 %)
	Descriptive analysis^a^ (15 %); Mixed methods (15 %)	Mixed methods (6 %)	Mixed methods (14 %)	Mixed methods (8 %)	Mixed methods (12 %)	Quantitative (21 %)
Document type						
Peer-review journal	86 %	79 %	87 %	79 %	86 %	95 %
Book	2 %	–	1 %	1 %	–	–
Book (edited volume)	7 %	17 %	10 %	14 %	12 %	5 %
Research report	5 %	4 %	2 %	6 %	2 %	–
Common features						
Multidimensionality	65 %	64 %	69 %	49 %	32 %	58
Dynamic elements	35 %	35 %	13 %	40 %	22 %	32 %
Agency elements	Implied	Implied	Implied	Implied	Implied	Implied
Relative elements	Implied	Implied	Implied	Implied	Implied	Implied

Context-orientated studies are excluded

^a^Relates to a theoretical argumentation based on a descriptive review of the literature

### Neighbourhood and community

Using domain-specific keywords, that included *neighbourhood*, *community*, *place*, *crime* and *safety*, and *social cohesion*, 116 texts were found, with seven dimensions identified. Neighbourhood context-oriented studies accounted for 61 publications (*n* = 61), and included research on neighbourhood influences and disability outcomes (e.g. Freedman et al. 2011; Marquet and Miralles-Guasch 2015). The remaining 55 documents addressed: social and relational aspects of place (*n* = 23); services, amenities and built environment (*n* = 22); place socio-economic aspects (*n* = 14); socio-political structures (*n* = 8); place-based policy (*n* = 5); and crime (*n* = 2). Studies on social and relational aspects (*n* = 23) concentrate on exclusion arising from deficient relational communities, declining social capital, reduced social participation and social cohesion (e.g. Burns et al. 2012; Walsh et al. 2012b; Buffel et al. 2014; Stoeckel and Litwin 2015). Work on services, amenities and built environment (*n* = 22) explores service retrenchment and reform, spatial inequalities in provision, and place-based transport issues (e.g. Shergold and Parkhurst 2012; Keene and Ruel 2013; Temelová and Slezáková 2014). Research on place socio-economic aspects (*n* = 14) focuses on spatially clustered poverty and deprivation (Scharf et al. 2005; Milbourne and Doheny 2012), and work on socio-political structures (*n* = 8) looks at the marginalisation of older residents and places from decision-making (Warburton et al. 2014; Burns et al. 2012). Research on place-based policy (n=5) explores how older adult residents are inadequately, or inappropriately recognised, by policy on, and implemented within, place. This work has in particular concentrated on offering critical analyses of age-friendly programmes (Scharlach and Lehning 2013; Keating et al. 2013; Walsh et al. 2014). Research on crime (*n* = 3; e.g. De Donder et al. 2005) had the lowest number of publications. Many texts fed into several of these dimensions, hence reported numbers do not sum to 116. There were also a number of cross-cutting themes. This included work on place belonging and the life course (*n* = 12; e.g. Russell et al. 1998; Walsh et al. 2012a), which could protect against or intensify exclusion. Research on change processes and macro forces (*n* = 10) illustrated how local shifts (e.g. out-migration) and macro-driven transformations (e.g. gentrification) can function to reduce social opportunities, alter service infrastructure and dilute place-based identity (*n* = 12; e.g. Phillipson 2007; Buffel et al. 2013; Walsh 2015). Urban and urban deprived contexts dominated the literature (*n* = 28), with rural settings considered less (*n* = 19). Within the 55 documents, over three-quarters (*n* = 43) reference social exclusion discourse in some form, and just over half (*n* = 30) consider exclusion within their main research question(s).

### Social relations

The review on social relations and exclusion identified 114 relevant studies. Employing such domain-specific keywords as *social relations*, *social connections*, *social resources*, *social network*, *loneliness*, and *isolation*, six different dimensions were identified within this body of work. Again, context-oriented papers on social relations accounted for the majority of studies (*n* = 45). This included work on topics such as correlates of loneliness (e.g. Dahlberg and McKee 2014) and network turnover (e.g. Conway et al. 2013). Of the remaining 69 texts, almost two-thirds (*n* = 45) referenced a social exclusion discourse with a third (*n* = 23) explicitly focused on exclusion. Twenty-nine publications considered social networks and support, exploring the mediating role of these resources and documenting mechanisms of exclusion arising from migration, deficient capacity for social capital generation, reduced formal supports, and social disadvantage (e.g. Ogg 2003; Ryser and Halseth 2011; Najsztub et al. 2015). Nineteen studies examined loneliness and isolation and, in particular, how risk factors around social location, social and health resources, educational attainment, economic hardship and changes over time in social resources can generate objective and subjective exclusionary impacts (e.g. Victor et al. 2005; Scharf and De Jong Gierveld 2008; Cloutier-Fisher et al. 2011; Victor and Bowling 2012; Burholt and Scharf 2014; De Jong Gierveld et al. 2015). Seventeen publications considered exclusion in relation to social opportunities and, in particular, their relationship to deficient financial resources, residential tenure, changing community socialisation, and choice constraints (e.g. O’Shea et al. 2012; Rozanova et al. 2012; Zhang and Zhang 2015). The dimensions of social relationship quality (*n* = 4; e.g. Yunong 2012), and conceptual work (*n* = 1) accounted for the fewest publications on exclusion-related topics. Cross-cutting themes relating to these dimensions included gender (*n* = 18; Russell and Porter 2003; Ziegler 2012), neighbourhood and community (*n* = 17; e.g. Boneham and Sixsmith 2006), immigrant groups (*n* = 9; e.g. Heikkinen 2011; Lee et al. 2014), individuals living alone and unmarried (*n* = 6; e.g. Banks et al. 2009), and family relations (*n* = 4; e.g. Ogg and Renaut 2012).

### Service, amenities and mobility

After full-text review, 106 studies across seven different dimensions were identified as relevant to exclusion in the services, amenities and mobility domain. Domain-specific keywords such as *service(s)*, *utilities*, *utilisation*, *transport*, and *mobility* were used to conduct the scoping review. In this domain, context-oriented papers accounted for only 16 studies, leaving 90 other publications. The dimensions of health and social care services, and transport and mobility represented the primary bodies of literature on old-age service exclusion, accounting for 34 and 20 texts, respectively. Research on the former concentrates on exclusion arising from such mechanisms as social and geographic location, market-modelled care reforms, poverty and accumulated disadvantage, discrimination and ageism, lack of cultural and language sensitivity, and failure to address needs of specific older adult sub-groups (e.g. Grenier and Guberman 2009; Parmar et al. 2014; Prada et al. 2015; Srakar et al. 2015). Exclusion in relation to transport and mobility focused on exclusionary processes stemming from lack of service flexibility, dependency on private transport options, disability and built environment access, and rural transport systems (e.g. Engels and Liu 2011; Giesel and Köhler 2015). The dimensions of area-based exclusion (e.g. Manthorpe et al. 2008), general services (e.g. Kendig et al. 2004) and information access and information and communication technologies (ICT) (e.g. Olphert and Damodaran 2013) were also well represented with 15, 11 and 10 texts, respectively. Work on conceptual underpinnings (*n* = 3; e.g. Simms 2004), and housing (*n* = 2; e.g. Peace and Holland 2001) attracted less research interest. Further thematic areas are identifiable across these seven dimensions. This includes work on gendered aspects of service exclusion (*n* = 22; e.g. Aronson and Neysmith 2001; Beaulaurier et al. 2014), and the experiences of specific groups of older people, such as members of LGBT (*n* = 4; e.g. McCann et al. 2013) and homeless communities (*n* = 3; e.g. Warnes and Crane, 2006), and persons with dementia (e.g. *n* = 4; e.g. O’Shea et al. 2015). While two-thirds of texts (*n* = 56) referred to exclusion, just under half (*n* = 39) had exclusion as a central focus.

### Material and financial resources

Ninety-five documents addressed exclusion from material and financial resources in later life. Using the domain-specific keywords of *poverty*, *low income*, *deprivation*, *material resources* and *financial resources*, six dimensions were identified. Context-oriented texts accounted for 23 studies, with an emphasis on topics such as socio-economic inequalities in health (e.g. Shaw et al. 2014) and impact of early-life circumstances (e.g. Shen and Zeng 2014). Of the remaining five dimensions and 72 texts, half of texts (*n* = 36) referred to a social exclusion discourse, while under a third (*n* = 20) concentrated on exclusion as the primary focus. Studies on poverty accounted for 28 publications and focused on determinants (such as: life-course multidimensional disadvantage; inadequate pension provisions; rural contexts; macro-economic recession conditions) and impacts (such as the onset of ill-health and disability) (e.g. Price 2006; Zaidi 2008; Milbourne and Doheny 2012; Patsios et al. 2012). Twenty-seven texts considered deprivation and material resources, exploring exclusionary mechanisms in relation to housing provision, gendered power relationships and deprived communities, and negative impacts with respect to social opportunities, and psychological and general well-being (e.g. Berthoud et al. 2009; Patsios 2014; Hunkler et al. 2015). The dimension of income, employment and pensions accounted for the next highest number of studies (*n* = 11; e.g. Dewilde 2012; Delfani et al. 2015). Fuel poverty (*n* = 3; e.g. Cotter et al. 2012) and conceptual elements (*n* = 3; e.g. Golant 2005) attracted the fewest publications. As with the other domains, several cross-cutting thematic areas were identifiable across the five dimensions and 72 texts. These included work on gender, focusing mainly on older women (*n* = 12; e.g. Ginn 1998; Ní Léime et al. 2015), life-course determinants of poverty and deprivation (*n* = 6; Heap et al. 2013), neighbourhood and community (*n* = 6; e.g. Scharf et al. 2005), experiences of ethnic minority groups (*n* = 6; e.g. Ahmad and Walker 1997; Lai 2011), and measurement (*n* = 4; e.g. O’Reilly 2002).

### Socio-cultural aspects of society

The domain-specific keywords of *burden*, *image*, *attitudes*, *symbolic*, *identity*, *cultural*, and *ageism* yielded 60 studies across five dimensions that were relevant to socio-cultural exclusion. Identity exclusion (i.e. reduction to one-dimensional identities) accounted for 23 publications, and focused on mechanisms in relation to social security individualisation; globalisation; social stratification and welfares states; failure to recognise gender, cultural and ethnic identities; and biomedical stigmatisation of age (e.g. Estes 2004; Twigg 2007; Wilińska and Henning 2011). Twenty-two texts considered symbolic and discourse exclusion (i.e. negative representations or constructions of ageing) and analysed exclusion emerging from: fixed social constructions of age; associations of active and successful ageing with work trajectories; and universality of frailty discourses; promotion of anti-ageing interventions (e.g. Biggs 2001; Gilleard and Higgs 2011; Laliberte 2015; Walsh et al. 2015). Work on ageism and age discrimination accounted for over one-fifth of all texts (*n* = 12; e.g. Duncan and Loretto 2004; Vitman et al. 2014; Carney and Gray 2015). Although only three documents explicitly considered the conceptualisation of socio-cultural exclusion (e.g. Jehoel-Gijsbers and Vrooman 2008), most publications contributed in some way to conceptual knowledge. Other thematic areas evident across dimensions included publications on gender (*n* = 9; e.g. Sabik 2014), employment and labour participation (*n* = 7; e.g. Taylor and Walker 1998), social policy and active ageing (*n* = 7; e.g. Biggs and Kimberley 2013), and members of particular older adult sub-groups, namely the LGBT community (*n* = 6; e.g. Harley et al. 2016) and ethnic minority groupings (*n* = 6; e.g. Zubair and Norris 2015). Over half of studies (*n* = 33) referred to social exclusion in their analysis, but just ten texts had an explicit focus on social exclusion.

### Civic participation

The search identified just 21 texts relevant to exclusion from civic participation. Using domain-specific keywords of: *civic*, *voting*, *volunteer*, *community responsibility*, *political* and *participation*, six dimensions were identified within this literature. Two publications were context orientated, addressing levels of political participation and determinants of social capital (e.g. Serrat et al. 2015). The remaining dimensions, encompassing 19 texts, focused on citizenship, conceptual underpinnings of exclusion from civic participation, general civic activities, volunteering and community responsibility, and voting and political participation. While no single dimension dominates, the greatest number of publications addressed the dimensions of voting and political participation, concentrating on deficient advocacy capacity and powerlessness (*n* = 5; e.g. Raymond and Grenier 2013); general civic activities, exploring health barriers and lack of state supports (*n* = 5; e.g. Hirshorn and Settersten 2013); and volunteering and community responsibility, analysing impediments to local governance participation and expectations for volunteering in later life (*n* = 4; e.g. Petriwskyj et al. 2012). Citizenship (*n* = 3; e.g. Craig 2004) and conceptualisation of civic exclusion (*n* = 2; e.g. Grenier and Guberman 2009) received less attention. Texts that addressed exclusion from civic participation in relation to neighbourhood and community (*n* = 4; e.g. Buffel et al. 2014), and healthy and active ageing policy and discourse (*n* = 3; e.g. Stephens et al. 2015) represent identifiable cross-cutting thematic areas. Almost two-thirds of publications (*n* = 11) recognised the multidimensionality of exclusion from civic participation, and one third (*n* = 6) acknowledged its dynamic nature.

## A framework for future study: existing knowledge and future directions

This article presents a two-stage scoping review that aimed to capture the ever-expanding, previously disparate, literatures on social exclusion in later life. Space constraints inhibit the detailed presentation of research on each domain. Nevertheless, the article provides a synthesis of knowledge on old-age exclusion. Our analysis draws together the disjointed evidence base concerning the disadvantage of older people, providing a foundation for the development of a coherent comprehensive discourse on old-age exclusion. Approximately, half of all reviewed documents did not refer explicitly to the construct of social exclusion. While this indicates commonalities between exclusion and other constructs of disadvantage, it also illustrates the power of the review as a means of unearthing knowledge that previously was not recognised as being part of a scientific understanding of old-age exclusion.

**Fig. 2:**
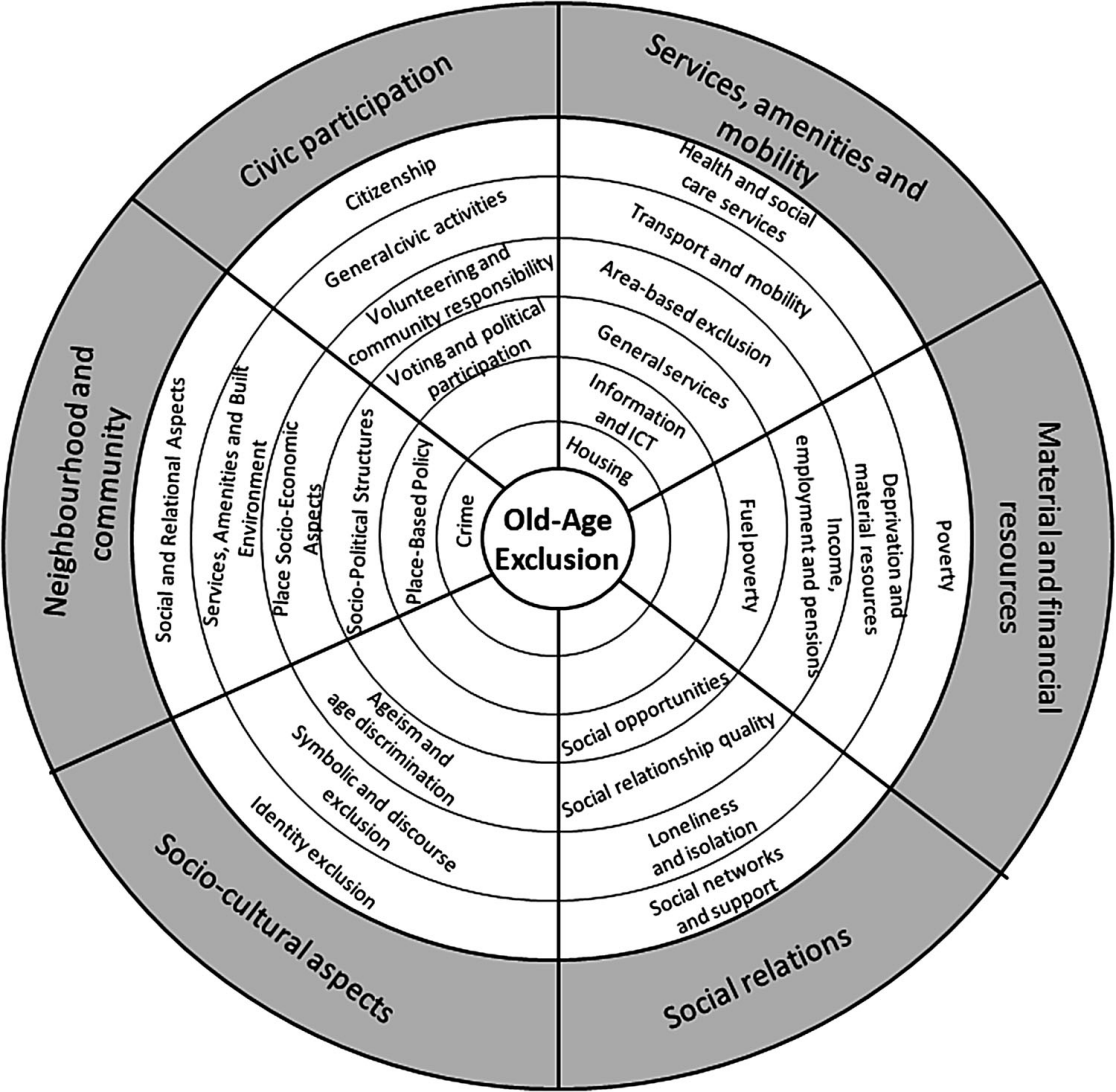
Old-age exclusion framework depicting interconnected domains and sub-dimensions

Figure 2 presents a framework, in the form of interconnected domains and sub-dimensions of old-age exclusion, derived from the assessment presented in the scoping review. This framework can serve as an orientating structure for future studies and analyses of multidimensional old-age exclusion. The figure illustrates the range of complex pathways to exclusion within each domain.

Although it is difficult to generalise beyond the contexts of specific forms and trajectories of disadvantage, it is possible to identify some broad operational and structural tenets of old-age exclusion across domains. In general, exclusionary channels appear to be multi-level, implicating not only the micro circumstances of individuals, but also typically meso- or macro-constructing forces (e.g. national employment policies combining with gendered social norms and community and household roles to exclude older women workers—Vera-Sanso 2012). These pathways are also multifaceted, impacting on multiple areas of life, e.g. transport exclusion leading to exclusion from health and social care services. Cross-cutting mechanisms of exclusion can be broadly pinpointed including geographic location and place context (e.g. Dwyer and Hardill 2011); social categorisations and marginalisation of particular groups (e.g. based on gender, ethnicity, income, and sexual orientation—McCann et al. 2013; Hunkler et al. 2015); life-course risk paths (e.g. Arber 2004), failure to recognise and address group-specific need (e.g. homeless older adults—Warnes and Crane, 2006; Beaulaurier et al. 2014); constrained choice and control (e.g. Rozanova et al. 2012); and diminished power (e.g. Raymond and Grenier 2013). The diminishing role of the state and increasing privatisation was also a notable cross-cutting exclusionary theme. Mostly evident in terms of individualisation of risk, service retrenchment and shifts in institutional policy, this act of exclusion involving the state was both direct and indirect in its agency and primarily implicated the domains of social relations (Walsh et al. 2012a), services (Grenier and Guberman 2009), and neighbourhood and community (e.g. Milbourne and Doheny 2012). In their own right, these cross-cutting mechanisms can represent outcomes and processes of exclusion embedded within complex pathways of disadvantage, with the influence of some of these mechanisms noted to be particularly difficult to unpack due to their interconnected nature (e.g. gender, social class, ethnicity and sexual orientation).

The question of how the ageing process itself intersects with such mechanisms is more difficult to answer. Thus what, if anything, makes old-age exclusion unique as a form of disadvantage, and specific to ageing? Three notable features can be discerned from the published material. First, there is a sense that exclusion can be accumulated over the course of older people’s lives, contributing to an increasing prevalence of exclusion into later life (e.g. Kneale 2012). Second, exclusionary mechanisms function as tipping points into precarity for ageing individuals, where older people have fewer opportunities and pathways to lift themselves out of exclusion (e.g. Scharf 2015). Third, in some cases, older people are more susceptible to exclusionary processes intersecting their lives and more vulnerable to the impacts of such exclusion mechanisms. This reflects the altered positioning of older adults with time, and the potential for age-related health declines, contracting social and support networks, and depleted income-generation opportunities (Jehoel-Gijsbers and Vrooman 2008; Walsh et al. 2012a).

Reflecting these summative synthesis points, and revisiting our working definition, we draw on the scoping review findings to propose a new definition of old-age exclusion:

Old-age exclusion involves interchanges between multi-level risk factors, processes and outcomes. Varying in form and degree across the older adult life course, its complexity, impact and prevalence are amplified by old-age vulnerabilities, accumulated disadvantage for some groups, and constrained opportunities to ameliorate exclusion. Old-age exclusion leads to inequities in choice and control, resources and relationships, and power and rights in key domains of neighbourhood and community; services, amenities and mobility; material and financial resources; social relations; socio-cultural aspects of society; and civic participation. Old-age exclusion implicates states, societies, communities and individuals.

The scoping review process has illuminated the nature and characteristics of the existing evidence-base. Our findings point to the relatively limited literature pertaining to old-age social exclusion. That stated, the scoping review points to a growing body of work on old-age exclusion, with 54 per cent of all (non-context orientated) papers published between 2010 and 2015. This increasing interest may be attributable to concerns surrounding global forces, such as economic uncertainty and the prevalence of individualisation of risk within policy discourses. It may also reflect the recognised value of social exclusion as an explanatory and flexible frame for understanding disadvantage in later life. Further, the review highlights the dominance of the UK as a source of research, reflecting the emergence of social exclusion as a significant social policy construct during the 1990s, and the UK’s longstanding research focus on ageing and structural disadvantage. However, with a growing prevalence of publications emanating from South America (Prada et al. 2015), North America (O’Rand 2006; Lee et al. 2014), Australasia (Winterton et al. 2014), Asia (Shirahase 2015) and Eastern Europe (Hrast et al. 2013), it is also evident that old-age exclusion is gathering traction as a global research topic.

Our findings demonstrate the general lack of conceptual work on exclusion of older people. The lack of work on unpacking the conceptual relationships between drivers and domains of exclusion is even more apparent (with the exception of Guberman and Lavoie 2004, Jehoel-Gijsbers and Vrooman 2008 and Walsh et al. 2012a). It is partly for this reason that it was necessary to include documents that contribute, in a broad way, to conceptual discourse of the construct (e.g. Scharf and Keating 2012; Börsch-Supan et al. 2015). Detailed conceptualisation in relation to each domain of exclusion is also generally lacking and is evidenced by the very small number of papers focusing on conceptual development across domains. Such a gap not only undermines the development of a critical understanding of old-age exclusion, but also limits our capacity to develop policy and practice interventions to reduce exclusion of older people. This may explain why exclusion has emerged redefined from Europe’s period of economic recession as a policy construct focused on single parents, young people and, principally, labour market participation.

The scoping review also identified areas that require further research. The most pressing area relates to the multidimensional construct of old-age exclusion itself. How the various experiences, processes and outcomes across domains and across the life course combine to generate exclusion remains a fundamental question. With respect to domain-specific work, Fig. 1 shows the dominance of neighbourhood and community; social relations; services, amenities and mobility; and material and financial resources in rank order. It is, however, more appropriate to exclude context-oriented publications altogether. This produces a different picture, one that is more reflective of traditionally dominant areas of research, with the following rank order: services, amenities and mobility (*n* = 90); material and financial resources (*n* = 72); social relations (*n* = 69); socio-cultural aspects (*n* = 60); neighbourhood and community (*n* = 55); and civic participation (*n* = 19). With environmental gerontology emerging rapidly as a core feature of research on age-related disadvantage, and with increased interest in spatially directed social policy (e.g. age-friendly communities; healthy cities), neighbourhood and community is likely to attract increasing attention in the study of social exclusion. Similarly, and in the context of a prevalent age-related burden discourse within European and international policy, and the proliferation of healthy and active ageing constructs, meaningful analyses and critiques of exclusion in civic and socio-cultural aspects of life are also likely to become more important.

Methodological gaps are similarly identifiable, with a relatively small proportion of mixed-method interdisciplinary work. There is also less of a focus than may have been expected on longitudinal inquiries, qualitative studies and life-course approaches.

The coverage of social categorisations, such as gender, ethnicity, income, and sexual orientation, was relatively weak. In some respects, this is likely to be connected to the difficulty in (quantitatively) isolating the directional associations of such categorisations, as noted within a number of operational- and empirically based conceptual frameworks (e.g. Barnes et al. 2006). Although covered to a greater extent within certain domains (services, amenities and mobility—McCann et al. 2013; Beaulaurier et al. 2014; material and financial resources—Ahmad and Walker 1997; Ní Léime et al. 2015; social relations—Ziegler 2012; Lee et al. 2014; socio-cultural aspects—Harley et al. 2016), and while gender attracts notably more attention than other categorisations, there are substantial gaps with respect to how the structural and societal positioning of all of these categorisations combine with ageing processes to produce exclusion. Deficits with respect to the exclusion of older people belonging to the LGBT community are especially apparent. Moreover, and illustrated again by the difficulties noted in the conceptual frameworks, work is required to disentangle the objective and subjective experiential intersections of these various categorisations across the ageing life course.

Few studies addressed exclusionary pathways of migrant groups (Heikkinen 2011; Victor et al. 2012). Given new and substantial migration flows occurring within and across world regions, analyses need to be increasingly framed through an age-related exclusionary lens. Emerging evidence indicates that large numbers of older people have migrated, with increasing recognition of older-adult forced migration patterns (Mölsä et al. 2014; Loi and Sundram 2014). Such trends raise complex questions around exclusion in each of the domains that are framed within pre-migration trauma, the ordeal of migration itself, post-migration stressors and competing notions of displacement and security (Mölsä et al. 2014; Walsh 2016).

The role of economic austerity and the global economic recession in generating exclusion received less consideration than may have been expected (Bonfatti et al. 2015; Scharf 2015). This was particularly surprising given the social, economic and cultural magnitude of the recession in Europe. There is the potential for financial insecurity, arising from the sharp contraction of pension wealth, decreased value of social benefits, and resource transfer to younger generations (Foster and Walker 2014), to impact on the lives of older adults in a multifaceted way. This extends beyond more complex pathways that implicate cuts to public expenditure in welfare, health and social systems that may increase older adult vulnerability. It is necessary to consider the longer-term exclusionary implications of such developments for Europe’s ageing societies.

As a significant contributor to the global disease burden, the fastest growing cause of disability (OECD 2015), and the potential for the condition itself and its care management to serve as an exclusionary mechanism (Österholm and Samuelsson 2015), it was also surprising that dementia did not feature strongly as a topic of exclusion research. Issues with respect to supporting people in their own communities, service access, and the societal positioning of older people with dementia certainly illustrate exclusion stemming from being diagnosed with this condition. While similar arguments could also be made for other conditions, this area requires future research to develop sociological understandings of related disadvantage.

## Conclusion

There are a number of limitations to this article. This includes its focus on English language studies only, difficulties in capturing all material outside of the exclusion discourse, and the limited space that prevents a detailed presentation of knowledge synthesis for each domain. Notwithstanding these issues, the article contributes to international debates on old-age exclusion. It unites disparate evidence on the exclusion of older people across topic areas and disciplines, and helps to inform a more coherent and comprehensive discourse on old-age exclusion. The presented framework harnesses this synthesis and offers a structure for guiding future empirical and conceptual work in this field of study. Old-age exclusion remains a fundamental challenge for ageing societies in Europe and beyond. It is only by sharing, synthesising and building upon state-of-the-art knowledge that we can begin to think about how to effectively and efficiently respond to this challenge.

## Electronic supplementary material

Below is the link to the electronic supplementary material.

Supplementary material 1 (DOCX 21 kb)

Supplementary material 2 (DOCX 25 kb)

Supplementary material 3 (DOCX 37 kb)

Supplementary material 4 (DOCX 97 kb)

## Footnotes

1 Social inclusion is an under-theorised topic and requires further work in its own right (Scharf and Keating 2012).
