# Supplementary material for: Social exclusion of older persons: a scoping review and conceptual framework
Source: Eur J Ageing. 2016 Oct 11;14(1):81–98. doi: 10.1007/s10433-016-0398-8 (PMC5550622; doi:10.1007/s10433-016-0398-8)
Supplement: Supplementary file 1 — Supplementary material 1 (DOCX 21 kb) [file 10433_2016_398_MOESM1_ESM.docx]

**Supplementary Material**

**Selected definitions of social exclusion**

| **Authors** | **Definition** | **Definition Emphasis** |
| --- | --- | --- |
| Xiberras (1993)  (cited in Silver 1995: 64) | The progressive rupture of the social and symbolic bonds – economic, institutional and meaningful – that normally attach each individual to society. | Multidimensional; Symbolic exchange; Distributional/relational elements;  Solidarity |
| Berghman (1995) | The denial or non-realisation of citizenship rights in four societal sub-systems – the labour market, the democratic and legal system, the welfare state and the family and community system. | Multidimensional; Citizenship; Social rights;  Distributional/relational elements |
| Duffy (1995) | An inability to participate effectively in economic, social, political or cultural life, alienation and distance from the mainstream society. | Multidimensional; Participation; Power relations |
| Gore (1995: 103) | Social exclusion refers, in broad terms, to a situation and a process which arises when individuals and groups are unable to achieve full membership in national society, in the sense that they cannot participate in the relationships and practices considered by that society to be customary and constitutive of membership. | Outcome/processes; Relativity; Relationships/practices; Participation |
| Rodgers (1995: 50) | The notion of exclusion links together both social rights and material deprivation. So it encompasses not only a lack of access to goods and services which underlie poverty and basic needs satisfaction, but also exclusion from security, from justice, from representation and from citizenship. | Multidimensional; Social rights; Material deprivation |
| Walker and Walker  (1997: 8) | The dynamic process of being shut out, fully or partially, from any of the social, economic, political or cultural systems which determine the social integration of a person in society. Social exclusion may, therefore, be seen as the denial (or non-realisation) of the civil, political and social rights of citizenship. | Multidimensional; Dynamics;  Distributional/relational elements; Rights; Integration; Agency; Processes |
| Mandipour et al. (1998) | A multidimensional process, in which various forms of exclusion are combined: participation in decision making and political processes, access to employment and material resources and integration into common cultural process. When combined, they create acute forms of exclusion that find a spatial manifestation in particular neighbourhoods. | Multidimensional; Distributional/relational elements; Participation; Process; Spatial elements |
| Burchardt (1999, 2000) | An individual is socially excluded if a) he or she is geographically resident in a society and b) he or she does not participate in the normal activities of that society. | Relativity; Participation |
| Peace (2001) | The collective processes that work to deprive people of access to opportunities and means, material or otherwise, to achieve well-being and security in the terms that are important to them. | Processes; Distributional/relational elements |
| Vleminckx and Berghman (2001: 46) | A concoction (or blend) of multidimensional and mutually reinforcing processes of deprivation, associated with progressive dissociation from social milieu, resulting in the isolation of individuals and groups from the mainstream of opportunities society has to offer. | Multidimensional; Dynamics; Processes;  Distributional/relational elements |
| Estivill (2003: 19) | Social exclusion may be understood as the accumulation of confluent processes with successive ruptures arising from the heart of the economy, politics and society, which gradually distances and places persons, groups, communities and territories in a position of inferiority in relation to centres of power, resources and prevailing values. | Multidimensional; Dynamics; Processes; Power relations |
| Silver and Miller (2003: 8) | Social exclusion is (1) multidimensional or socioeconomic, and encompasses collective as well as individual resources, (2) dynamic or processual, along a trajectory between full integration and multiple exclusions, (3) relational, in that exclusion entails social distance or isolation, rejection, humiliation, lack of social support networks, and denial of participation, (4) active, in that there is a clear agency doing the excluding, and (5) relative to context. | Multidimensional; Dynamics; Distributional/relational elements; Processes; Relativity |
| Levitas et al. (2007) | Social exclusion is a complex and multi-dimensional process. It involves the lack or denial of resources, rights, goods and services, and the inability to participate in the normal relationships and activities, available to the majority of people in a society, whether in economic, social, cultural or political arenas. It affects both the quality of life of individuals and the equity and cohesion of society as a whole. | Multidimensional; Process; Participation;  Distributional/relational elements;  Relativity; Social rights; Cohesion |
| Billette and Lavoie (2010) cited in Burns et al. (2012: 2) | A process of non-acknowledgement and deprivation of rights and resources of certain segments of the population that takes the shape of power dynamics between groups with divergent visions and interests. Such processes result in inequalities and lead eventually to isolation from society in multiple dimensions. | Multidimensional; Distributional/relational elements; Power dynamics; Rights; Diversity |

**References**

1. Berghman J (1995) Social exclusion in Europe: policy context and analytical framework. In: Room G. (ed) Beyond the Threshold: The measurement and analysis of social exclusion, Policy Press, Bristol, pp 10-28
2. Billette V, Lavoie JP (2010) Introduction. Vieillissements, exclusions sociales et solidarités. In: Charpentier MN, Billette V, Lavoie JP, Grenier A, Olazabal I (eds) Vieillir au Pluriel. Perspectives Sociales, Presses de l'Université du Québec, Québec, pp 1-22
3. Burchardt T (2000) Social exclusion: concepts and evidence. In: Gordon D, Townsend P (eds) Breadline Europe: The measurement of poverty. Bristol Policy, Bristol, pp 385-406
4. Burns VF, Lavoie JP, Rose D (2012) Revisiting the role of neighbourhood change in social exclusion and inclusion of older people. Journal of Aging Research 2012:148287-148287
5. Duffy K (1995) Social exclusion and human dignity in Europe. Council of Europe, Strasbourg
6. Estivill J (2003) Concepts and strategies for combating social exclusion: An overview. International Labour Organization
7. Gore C (1995) Social exclusion and social change: Insights in the African literature. In: Rodgers G, Gore C, Figueiredo J (eds) Social Exclusion: Rhetoric, Reality, Responses. International Institute for Labour Studies. ILO Publications, Geneva, pp 103-117
8. Levitas R, Pantazis C, Fahmy E et al. (2007) The multi-dimensional analysis of social exclusion: Cabinet Office London
9. Madanipour A, Cars G et al. (1998) Social exclusion in European cities: processes, experiences, and responses. Jessica Kingsley Publishers, London
10. Peace R (2001) Social exclusion: A concept in need of definition? Soc Policy J N Z 16:17-36
11. Rodgers G (1995) What is special about a social exclusion approach? In: Rodgers G, Gore C, Figueiredo J (eds) Social exclusion: Rhetoric, reality, responses. International Institute for Labour Studies. ILO Publications, Geneva, pp 43-55
12. Silver H (1995) Reconceptualising social disadvantage: three paradigms of social exclusion. in Rodgers G, Gore C, and Figueiredo JB (eds) Social exclusion: Rhetoric, reality, responses. Geneva: International Labour Organization
13. Silver H, Miller S (2003) Social exclusion. The european approach to social disadvantage. Indicators 2:1-17
14. Vleminckx K, Berghman J (2001) Social exclusion and the welfare state: an overview of conceptual issues and policy implications. In: Mayes D, Berghman J, Salais R (eds) Social exclusion and European policy. Edward Elgar, Northampton, pp 27-46
15. Walker A, Walker C (1997) Britain divided: The growth of social exclusion in the 1980s and 1990s. CPAG, London
16. Xiberras M (1993) Les théories de l’exclusion. Meridiens Klincksieck, Paris
