# Supplementary material for: Social exclusion of older persons: a scoping review and conceptual framework
Source: Eur J Ageing. 2016 Oct 11;14(1):81–98. doi: 10.1007/s10433-016-0398-8 (PMC5550622; doi:10.1007/s10433-016-0398-8)
Supplement: Supplementary file 2 — Supplementary material 2 (DOCX 25 kb) [file 10433_2016_398_MOESM2_ESM.docx]

**Supplementary Material**

**Flow diagram for stage one of scoping review**

Stage One

search results

(1997-2015)

Relevant after title review

n=227

Relevant after full text review

Included for charting

n=25

Relevant after abstract review

n=90

Original conceptualisations

n=8 Original conceptualisations

n=8

Reference list search

n=2

Relevant to general conceptualisations

n=12

Relevant to related concepts

n=5
