# Supplementary material for: Social exclusion of older persons: a scoping review and conceptual framework
Source: Eur J Ageing. 2016 Oct 11;14(1):81–98. doi: 10.1007/s10433-016-0398-8 (PMC5550622; doi:10.1007/s10433-016-0398-8)
Supplement: Supplementary file 3 — Supplementary material 3 (DOCX 37 kb) [file 10433_2016_398_MOESM3_ESM.docx]

**Supplementary Material**

**Stage one findings: conceptual frameworks of social exclusion of older persons**

**References**

1. Barnes M, Blom A, Cox K et al. (2006) The social exclusion of older people: evidence from the first wave of the English Longitudinal Study of Ageing (ELSA): final Report. Office for the Deputy of Prime Minister
2. Börsch-Supan A, Kneip T, Litwin H, Myck M, Weber G (2015) SHARE: a European policy device for inclusive ageing societies. In: Börsch-Supan A, Kneip T, Litwin H, Myck M, Weber G (eds) Ageing in Europe - supporting policies for an inclusive society. DE GRUYTER, pp 1-22
3. Cavalli S, Bickel JF (2007) Exclusion in Very Old Age. International Journal of Ageing and Later Life 2:9-31
4. Dannefer D (2003) Cumulative advantage/disadvantage and the life course: Cross-fertilizing age and social science theory. J Gerontol B-Psychol 58:327-337
5. Dewilde C (2003) A life-course perspective on social exclusion and poverty. Brit J Sociol 54:109-128
6. Feng W (2003) Social exclusion of the elderly in China: one potential challenge resulting from the rapid population ageing in Demographic Change and Local Development: Shrinkage, Regeneration and Social Dynamics. OECD
7. Grundy E (2006) Ageing and vulnerable elderly people: European perspectives. Ageing Soc 26:105-134
8. Guberman N, Lavoie JP (2004) Equipe Vies: Framework on Social Exclusion. Centre de recherche et d’expertise de gérontologie sociale – CAU/CSSS Cavendish, Montréal, QC
9. Hoff A (2008) Tackling poverty and social exclusion of older people: Lessons from Europe: Oxford Institute of Ageing, Oxford
10. Hrast MF, Mrak AK, Rakar T (2013) Social exclusion of elderly in Central and Eastern Europe. Int J Soc Econ 40:971-989
11. Jehoel-Gijsbers G, Vrooman J (2008) Social Exclusion of the Elderly. Centre for European Policy Studies, European Network of Policy Research Institutes
12. Kneale D (2012) Is social exclusion still important for older people? The International Longevity Centre–UK Report
13. Lee Y, Hong PYP, Harm Y (2014) Poverty among Korean immigrant older adults: Examining the effects of Social Exclusion. J Soc Serv Res 40:385-401
14. Lui CW, Warburton J, Winterton R et al. (2011) Critical reflections on a social inclusion approach for an ageing Australia. Aust Soc Work 64:266-282
15. Myck M, Najsztub M, Oczkowska M (2015) Measuring social deprivation and social exclusion. In: Börsch-Supan A, Kneip T, Litwin H, Myck M, Weber G (eds) Ageing in Europe - supporting policies for an inclusive society. DE GRUYTER, pp 67-78
16. Ogg J (2005) Social exclusion and insecurity among older Europeans: the influence of welfare regimes. Ageing Soc 25:69-90
17. Patsios D (2000) Poverty and social exclusion amongst the elderly. University of Bristol, Townsend Centre
18. Scharf T (2015) Between inclusion and exclusion in later life. In: Walsh K, Carney G, Ní Léime Á (eds) Ageing through Austerity: Critical perspectives from Ireland. Policy Press, Bristol, pp 113-130
19. Scharf T, Keating N (2012) Social exclusion in later life: a global challenge. In: Scharf T, Keating N (eds) From Exclusion to Inclusion in Old Age: A Global Challenge. The Policy Press, Bristol, pp 1-16
20. Scharf T, Bartlam B (2008) Ageing and social exclusion in rural communities. In: Keating N (ed) Rural ageing: a good place to grow old? Policy Press, Bristol, pp 97-108
21. Scharf T, Phillipson C and Smith AE. (2005) Multiple exclusion and quality of life among excluded older people in disadvantaged neighbourhoods. Social Exclusion Unit, Minister OotDP, London
22. Scharf T, Phillipson C, Kingston P et al. (2001) Social exclusion and older people: exploring the connections. Education and Ageing 16:303-320
23. Schröder-Butterfill E, Marianti R (2006) A framework for understanding old-age vulnerabilities. Ageing Soc 26:9-35
24. Walsh K, O’Shea E, Scharf T (2012) Social Exclusion and Ageing in Diverse Rural Communities: Findings of a cross border study in Ireland and Northern Ireland. Irish Centre for Social Gerontology
25. Warburton J, Ng SH, Shardlow SM (2013) Social inclusion in an ageing world: Introduction to the special issue. Ageing Soc 33:1-15
