# Supplementary material for: Social exclusion of older persons: a scoping review and conceptual framework
Source: Eur J Ageing. 2016 Oct 11;14(1):81–98. doi: 10.1007/s10433-016-0398-8 (PMC5550622; doi:10.1007/s10433-016-0398-8)
Supplement: Supplementary file 4 — Supplementary material 4 (DOCX 97 kb) [file 10433_2016_398_MOESM4_ESM.docx]

**Supplementary Material**

**Stage two findings: domains of social exclusion of older persons**

**References**

1. Abbott P, Sapsford R (2005) Living on the margins: Older people, place and social exclusion. Policy Studies 26:29-46
2. Ahern A, Hine J. (2012) Rural transport – Valuing the mobility of older people. Res Trans E 34:27-34
3. Ahmad WIU, Walker R (1997) Asian older people: housing, health and access to services. Ageing Soc 17:141-165
4. Akincigil A, Crystal S, Sambamoorthi U et al. (2003) Diagnosis and treatment of depression in the elderly medicare population: predictors, disparities, and trends. J Am Geriatr Soc 51:1718-1728
5. Al Hazzouri AZ, Haan MN, Osypuk T et al. (2011) Neighborhood socioeconomic context and cognitive decline among older Mexican Americans: Results from the Sacramento Area Latino Study on Aging. Am J Epidemiol 174:423-431
6. Allan D, Cloutier-Fsher D (2006) Health service utilization among older adults in British Columbia: Making sense of geography. Can J Aging 25:219-232
7. Alwan N, Wilkinson M, Birks D et al. (2007) Do standard measures of deprivation reflect health inequalities in older people? J Public Health Pol 28:356-362
8. Andrews G, Parkhurst G, Susilo YO et al. (2012) The grey escape: investigating older people's use of the free bus pass. Transport Plan Techn 35:3-15
9. Aneshensel CS, Wight RG, Miller-Martinez D, et al. (2007) Urban neighborhoods and depressive symptoms among older adults. J Gerontol B-Psychol 62:52-59
10. Arber S (2004) Gender, marital status, and ageing: linking material, health, and social resources. J Aging Stud 18:91-108
11. Aronson J, Neysmith SM (2001) Manufacturing social exclusion in the home care market. Can Public Pol 27:151-165
12. Aronson J (2006) Silenced complaints, suppressed expectations: The cumulative effects of home care rationing. Int J Health Serv 36:535-556
13. Arun O, Cakiroglu-Cevik A (2013) Quality of life in an ageing society: A comparative analysis of age cohorts in Turkey. Z Gerontol Geriatr 46:734-739
14. Auchincloss AH, van Nostrand JF, Ronsaville D (2001) Access to health care for older persons in the United States. J Aging Health 13:329-354
15. Avlund K, Damsgaard MT, Holstein BE (1998) Social relations and mortality. An eleven year follow-up study of 70-year-old men and women in Denmark. Soc Sci Med 47:635-643
16. Ayalon L, Shiovitz-Ezra S, Palgi Y (2013) Associations of loneliness in older married men and women. Aging Ment Health 17:33-39
17. Banks L, Haynes P, Hill M (2009) Living in single person households and the risk of isolation in later life. International Journal of Ageing and Later Life 4:55-86
18. Barrett AE, Pai M, Redmond R (2012) “It's your badge of inclusion”: The Red Hat Society as a gendered subculture of aging. J Aging Stud 26:527-538
19. Barrett GA, McGoldrick C (2013) Narratives of (in) active ageing in poor deprived areas of Liverpool, UK. International Journal of Sociology and Social Policy 33:347-356
20. Barrett J (2005) Support and information needs of older and disabled older people in the UK. Appl Ergon 36:177-183
21. Baxter K, Glendinning C (2011) Making choices about support services: disabled adults' and older people's use of information. Health Soc Care Comm 19:272-279
22. Beard RL, Knauss J, Moyer D (2009) Managing disability and enjoying life: How we reframe dementia through personal narratives. J Aging Stud 23:227-235
23. Beaulaurier R, Fortuna K, Lind D et al. (2014) Attitudes and stereotypes regarding older women and HIV risk. J Woman Aging 26:351-368
24. Beckett M, Goldman N, Weinstein M et al. (2002) Social environment, life challenge, and health among the elderly in Taiwan. Soc Sci Med 55:191-209
25. Beech R and Murray M (2013) Social engagement and healthy ageing in disadvantaged communities. Quality in Ageing and Older Adults 14:12-24
26. Berthoud R, Blekesaune M, Hancock R (2009) Ageing, income and living standards: Evidence from the British Household Panel Survey. Ageing Soc 29:1105-1122
27. Bertoni M, Cavapozzi D, Celidoni M, Trevisan E (2015) Assessing the material deprivation of older Europeans. In: Börsch-Supan A, Kneip T, Litwin H, Myck M, Weber G (eds) Ageing in Europe - supporting policies for an inclusive society. DE GRUYTER, pp 49-56
28. Bertoni M, Cavapozzi D, Celidoni M, Trevisan E (2015) Development and validation of a material deprivation index. In: Börsch-Supan A, Kneip T, Litwin H, Myck M, Weber G (eds) Ageing in Europe - supporting policies for an inclusive society. DE GRUYTER, pp 57-66
29. Bertoni M, Celidoni M, Weber G, Kneip T (2015) Does hearing impairment lead to social exclusion? In: Börsch-Supan A, Kneip T, Litwin H, Myck M, Weber G (eds) Ageing in Europe - supporting policies for an inclusive society. DE GRUYTER, pp 93-102
30. Biggs S, Kimberley H (2013) Adult ageing and social policy: New risks to identity. Social Policy and Society 12:287-297
31. Biggs S (2001) Toward critical narrativity: Stories of aging in contemporary social policy. J Aging Stud 15:303-316
32. Bishop AJ, Martin P (2007) The indirect influence of educational attainment on loneliness among unmarried older adults. Educ Gerontol 33:897-917
33. Blakemore K (2000) Health and social care needs in minority communities: An over-problematized issue? Health Soc Care Comm 8:22-30
34. Bond J, Corner L, Graham R (2004) Social science theory on dementia research: normal ageing, cultural representation and social exclusion. In: Innes A, Archibald C, Murphy C (eds) Dementia and social inclusion: Marginalised groups and marginalised areas of dementia research, care and practice. Jessica Kingsley Publishers, London, pp220-236.
35. Boneham MA, Sixsmith JA (2006) The voices of older women in a disadvantaged community: Issues of Health and Social Capital. Soc Sci Med 62:269-279
36. Bonfatti A, Celidoni M, Weber G, Börsch-Supan A (2015) Coping with risks during the Great Recession. In: Börsch-Supan A, Kneip T, Litwin H, Myck M, Weber G (eds) Ageing in Europe - supporting policies for an inclusive society. DE GRUYTER, pp 225-234
37. Bowling A, Stafford M (2007) How do objective and subjective assessments of neighbourhood influence social and physical functioning in older age? Findings from a British survey of ageing. Soc Sci Med 64:2533-2549
38. Boyle G (2010) Social policy for people with dementia in England: Promoting human rights? Health Soc Care Comm 18:511-519
39. Breeze E, Jones D, Wilkinson P et al. (2005) Area deprivation, social class, and quality of life among people aged 75 years and over in Britain. Int J Epidemiol 34:276-283
40. Breheny M, Stephens C (2010) Ageing in a material world. New Zeal J Psychol 39:41-48
41. Brotman S, Ferrer I, Sussman T et al. (2015) Access and equity in the design and delivery of health and social care to LGBTQ older adults: A Canadian perspective. In: Orel NA (ed), US: American Psychological Association, Washington DC, pp 111-140
42. Buffel T, De Donder L, Phillipson C et al. (2014) Social participation among older adults living in medium-sized cities in Belgium: The role of neighbourhood perceptions. Health Promot Int 29:655-668
43. Buffel T, Phillipson C, Scharf T (2012) Ageing in urban environments: Developing 'age-friendly' cities. Crit Soc Policy 32:597-617
44. Buffel T, Phillipson C, Scharf T (2013) Experiences of neighbourhood exclusion and inclusion among older people living in deprived inner-city areas in Belgium and England. Ageing Soc 33:89-109
45. Burge S, Street D (2010) Advantage and choice: Social relationships and staff assistance in assisted living. J Gerontol B-Psychol 65: 358-369
46. Burholt V, Scharf T (2014) Poor health and loneliness in later life: The role of depressive symptoms, social resources, and rural environments. J Gerontol B-Psychol 69:311-324
47. Burholt V, Windle G (2006) Keeping warm? Self-reported housing and home energy efficiency factors impacting on older people heating homes in North Wales. Energ Policy 34:1198-1208
48. Burholt V, Windle G (2006) The material resources and well-being of older people. Joseph Rowntree Foundation, Bangor
49. Burns VF, Lavoie JP, Rose D (2012) Revisiting the role of neighbourhood change in social exclusion and inclusion of older people. Journal of Aging Research 2012:148287-148287
50. Buys DR, Borch C, Drentea P et al. (2013) Physical impairment is associated with nursing home admission for older adults in disadvantaged but not other neighborhoods: Results from the UAB Study of Aging. Gerontologist 53:641-653
51. Buys DR, Howard VJ, McClure LA et al. (2015) Association between neighborhood disadvantage and hypertension prevalence, awareness, treatment, and control in older adults: results from the University of Alabama at Birmingham Study of Aging. Am J Public Health 105:1181-1188
52. Byles JE, Leigh L, Vo K et al. (2015) Life space and mental health: A study of older community-dwelling persons in Australia. Aging Ment Health 19:98-106
53. Calasanti T (2004) Feminist gerontology and old men. J Gerontol B-Psychol 59:305-314
54. Callander EJ, Schofield DJ, Shrestha RN (2012) Multiple disadvantages among older citizens: What a multidimensional measure of poverty can show. Journal of Aging and Social Policy 24:368-383
55. Carney GM (2015) Citizenship in an age of austerity: towards a constructive politics of ageing. In: Walsh K, Carney G, Ní Léime Á (eds) Ageing through austerity: Critical perspectives from Ireland. Policy Press, Bristol, pp 31-46
56. Carney GM, Gray M (2015) Unmasking the ‘elderly mystique’: Why it is time to make the personal political in ageing research. J Aging Stud 35:123-134
57. Carpiac-Claver ML, Levy-Storms L (2007) In a manner of speaking: Communication between nurse aides and older adults in long-term care settings. Health Commun 22:59-67
58. Carrino L, Orso CE (2015) Eligibility regulations and formal home-care utilisation among the vulnerable older people in SHARE Wave 5. In: Börsch-Supan A, Kneip T, Litwin H, Myck M, Weber G (eds) Ageing in Europe - supporting policies for an inclusive society. DE GRUYTER, pp 343-352
59. Chen Y, Hicks A, While AE (2014) Loneliness and social support of older people living alone in a county of Shanghai, China. Health Soc Care Comm 22:429-438
60. Chen Y, Hicks A, While AE (2014) Quality of life and related factors: A questionnaire survey of older people living alone in Mainland China. Qual Life Res 23:1593-1602
61. Cheng YH, Chi I, Boey KW et al. (2002) Self-rated economic condition and the health of elderly persons in Hong Kong. Soc Sci Med 55:1415-1424
62. Chiu S, Ngan R (1999) Employment of Chinese older workers in Hong Kong: cultural myths, discrimination and opportunities. Ageing International 25:14-30
63. Choi N (2011) Relationship between health service use and health information technology use among older adults: Analysis of the US National Health Interview Survey. J Med Internet Res 13
64. Chui E (2000) Boom the city, doom the elderly: housing problems of elderly affected by urban redevelopment in Hong Kong. Hallym International Journal of Aging 2:119-134
65. Chui E (2001) Doomed elderly people in a booming city: Urban redevelopment and housing problems of elderly people in Hong Kong. Hous Theory Soc 18:158-166
66. Clough B, Brazier M (2014) Never too old for health and human rights? Medical Law International 14:133-156
67. Cloutier-Fisher D, Kobayashi K, Smith A (2011) The subjective dimension of social isolation: A qualitative investigation of older adults' experiences in small social support networks. J Aging Stud 25:407-414
68. Conway F, Magai C, Jones S et al. (2013) A six-year follow-up study of social network changes among African-American, Caribbean, and U.S.-Born Caucasian urban older adults. Int J Aging Hum Dev 76:1-27
69. Cornwell B, Schumm LP, Laumann EO (2008) The social connectedness of older adults: A national profile. Am Sociol Rev 73:185-203
70. Cornwell B (2015) Social disadvantage and network turnover. J Gerontol B-Psychol 70:132-142
71. Cornwell EY and Waite LJ (2009) Social disconnectedness, perceived isolation, and health among older adults. J Health Soc Behav 50:31-48
72. Cotter N, Monahan E, McAvoy H et al. (2012) Coping with the cold - exploring relationships between cold housing, health and social wellbeing in a sample of older people in Ireland. Quality in Ageing and Older Adults 13:38-47
73. Countouris M, Gilmore S, Yonas M (2014) Exploring the impact of a community hospital closure on older adults: A focus group study. Health Place 26:143-148
74. Craig G (2004) Citizenship, exclusion and older people. J Soc Policy 33:95-114
75. Cramm JM and Nieboer AP (2013) Relationships between frailty, neighborhood security, social cohesion and sense of belonging among community-dwelling older people. Geriatr Gerontol Int 13:759-763
76. Crane M, Warnes AM (2000) Policy and service responses to rough sleeping among older people. J Soc Policy 29:21-36
77. Crane M, Warnes AM (2005) Responding to the needs of older homeless people: The effectiveness and limitations of British services. Innovation-Abingdon 18:137-152
78. Crane M, Warnes AM (2012) Homeless people: Older people. In: Smith SJ (ed) International encyclopedia of housing and home. Elsevier, San Diego, pp 104-110
79. Crane M, Byrne K, Fu R et al. (2005) The causes of homelessness in later life: Findings from a 3-nation study. J Gerontol B-Psychol 60:152-159
80. Croda E (2015) Pain and social exclusion among the European older people. In: Börsch-Supan A, Kneip T, Litwin H, Myck M, Weber G (eds) Ageing in Europe - supporting policies for an inclusive society. DE GRUYTER, pp 313-320
81. Cuddy AJ, Norton MI, Fiske ST (2005) This old stereotype: The pervasiveness and persistence of the elderly stereotype. J Soc Issues 61:267-285
82. Dahlberg L, McKee KJ (2014) Correlates of social and emotional loneliness in older people: evidence from an English community study. Aging Ment Health 18:504-514
83. Dai H, Jia G, Liu K (2015) Health-related quality of life and related factors among elderly people in Jinzhou, China: a cross-sectional study. Public Health 129:667-673
84. Davey JA (2007) Older people and transport: coping without a car. Ageing Soc 27:49-66
85. Day R (2010) Environmental justice and older age: Consideration of a qualitative neighbourhood-based study. Environ Plann A 42:2658-2673
86. De Donder L, Verté D, Messelis E (2005) Fear of crime and elderly people: Key factors that determine fear of crime among elderly people in West Flanders. Ageing International 30:363-376
87. De Jong Gierveld J, Van der Pas S, Keating N (2015) Loneliness of older immigrant groups in Canada: Effects of ethnic-cultural background. Journal of Cross-Cultural Gerontology 30:251-268
88. Deeming C (2011) Food and nutrition security at risk in later life: Evidence from the United Kingdom expenditure & food survey. J Soc Policy 40:471-492
89. Deindl C, Brandt M, Litwin H (2015) Social exclusion and support between generations. In: Börsch-Supan A, Kneip T, Litwin H, Myck M, Weber G (eds) Ageing in Europe - supporting policies for an inclusive society. DE GRUYTER, pp 161-168
90. Delfani N, De Deken J, Dewilde C (2015) Poor because of low pensions or expensive housing? The combined impact of pension and housing systems on poverty among the elderly. International Journal of Housing Policy 15:260-284
91. Dent OE, Broe GA, Creasey H et al. (1999) Transportation Needs of Community-living Older People in Sydney. Aust J Ageing 18:186-190
92. Denvir C, Balmer NJ, Pleasence P (2014) Portal or pot hole? Exploring how older people use the 'information superhighway' for advice relating to problems with a legal dimension. Ageing Soc 34:670-699
93. Dewilde C, Raeymaeckers P (2008) The trade-off between home-ownership and pensions: Individual and institutional determinants of old-age poverty. Ageing Soc 28:805-830
94. Dewilde C (2012) Lifecourse determinants and incomes in retirement: Belgium and the United Kingdom compared. Ageing Soc 32:587-615
95. DiNapoli EA, Wu B, Scogin F (2014) Social isolation and cognitive function in Appalachian older adults. Res Aging 36:161-179
96. Dodge BA (2008) Primary healthcare for older people: a participatory study in 5 Asian countries. HelpAge International, Chiang Mai
97. Duncan C, Loretto W (2004) Never the right age? Gender and age‐based discrimination in employment. Gend Work Organ 11:95-115
98. Duncan C (2003) Assessing anti-ageism routes to older worker re-engagement. Work Employ Soc 17:101-120
99. Dwyer P, Hardill I (2011) Promoting social inclusion? The impact of village services on the lives of older people living in rural England. Ageing Soc 31:243-264
100. Elliott J, Gale CR, Parsons S et al. (2014) Neighbourhood cohesion and mental wellbeing among older adults: A mixed methods approach. Soc Sci Med 107:44-51
101. Ellwardt L, Aartsen M, Deeg D et al. (2013) Does loneliness mediate the relation between social support and cognitive functioning in later life? Soc Sci Med 98:116-124
102. Engels B, Liu G-J (2011) Social exclusion, location and transport disadvantage amongst non-driving seniors in a Melbourne municipality, Australia. J Transp Geogr 19:984-996
103. Eschbach K, Ostir GV, Patel KV et al. (2004) Neighborhood context and mortality among older Mexican Americans: is there a barrio advantage? Am J Public Health 94:1807-1812
104. Estes CL (2004) Social security privatization and older women: a feminist political economy perspective. J Aging Stud 18:9-26
105. Evandrou M, Falkingham J, Feng Z et al. (2014) Individual and province inequalities in health among older people in China: Evidence and policy implications. Health Place 30:134-144
106. Ferreira M (2006) Differential impact of social-pension income on household poverty alleviation in three South African ethnic groups. Ageing Soc 26:337-354
107. Fokkema T, Naderi R (2013) Differences in late-life loneliness: a comparison between Turkish and native-born older adults in Germany. Eur J Ageing 10:289-300
108. Fokkema T, De Jong Gierveld J, Dykstra PA (2012) Cross-national differences in older adult loneliness. Journal of Psychology: Interdisciplinary and Applied 146:201-228
109. Fontaine R, Pino M, Jean-Baptiste M, Philibert A, Briant N, Joël ME (2015) Older adults living with cognitive and mobility-related limitations: social deprivation and forms of care received. In: Börsch-Supan A, Kneip T, Litwin H, Myck M, Weber G (eds) Ageing in Europe - supporting policies for an inclusive society. DE GRUYTER, pp 103-114
110. Foster L (2011) Older people, pensions and poverty: An issue for social workers? Int Soc Work 54:344-360
111. Fox KR, Hillsdon M, Sharp D et al. (2011) Neighbourhood deprivation and physical activity in UK older adults. Health Place 17:633-640
112. Franzese F (2015) Slipping into poverty: effects on mental and physical health. In: Börsch-Supan A, Kneip T, Litwin H, Myck M, Weber G (eds) Ageing in Europe - supporting policies for an inclusive society. DE GRUYTER, pp 139-148
113. Freedman VA, Grafova IB, Rogowski J (2011) Neighborhoods and chronic disease onset in later life. Am J Public Health 101:79-86
114. Freedman VA, Grafova IB, Schoeni RF et al. (2008) Neighborhoods and disability in later life. Soc Sci Med 66:2253-2267
115. Fristedt S, Dahl AK, Wretstrand A et al. (2014) Changes in community mobility in older men and women. A 13-year prospective study. PLoS ONE 9
116. Fry CL (2005) Globalization and the experiences of aging. Gerontology and Geriatrics Education 26:9-22
117. Gadalla TM (2010) The role of mastery and social support in the association between life stressors and psychological distress in older Canadians. J Gerontol Soc Work 53:512-530
118. Galčanová L, Sýkorová D (2015) Socio-spatial aspects of ageing in an urban context: An example from three Czech Republic cities. Ageing Soc 35:1200-1220
119. Gale CR, Dennison EM, Cooper C et al. (2011) Neighbourhood environment and positive mental health in older people: The Hertfordshire Cohort Study. Health Place 17:867-874
120. Gerst-Emerson K, Shovali TE, Markides KS (2014) Loneliness among very old Mexican Americans: Findings from the Hispanic established populations epidemiologic studies of the elderly. Arch Gerontol Geriat 59:145-149
121. Giarchi GG (2006) Older people 'on the edge' in the countrysides of Europe. Soc Policy Admin 40:705-721
122. Giesel F, Köhler K (2015) How poverty restricts elderly Germans’ everyday travel. European Transport Research Review 7
123. Giesel F, Rahn C (2015) Everyday life in the suburbs of Berlin: Consequences for the social participation of aged men and women. J Women Aging 27:330-351
124. Gilleard C, Higgs P (2011) Ageing abjection and embodiment in the fourth age. J Aging Stud 25:135-142
125. Ginn J (1998) Older women in Europe: East follows West in the feminization of poverty? Ageing International 24:101-122
126. Glendinning C (2008) Increasing choice and control for older and disabled people: A critical review of new developments in England. Soc Policy Admin 42:451-469
127. Golant SM (2005) Supportive housing for frail, low-income older adults: identifying need and allocating resources. Generations 29:37-43
128. Goli S, Singh L, Jain K et al. (2014) Socioeconomic determinants of health inequalities among the older population in India: a decomposition analysis. Journal of cross-cultural gerontology 29:353-369
129. Grant TL, Edwards N, Sveistrup H et al. (2010) Inequitable walking conditions among older people: examining the interrelationship of neighbourhood socio-economic status and urban form using a comparative case study. Bmc Public Health 10:677
130. Gray A (2009) The social capital of older people. Ageing Soc 29:5-31
131. Green J, Jones A, Roberts H (2012) More than A to B: the role of free bus travel for the mobility and wellbeing of older citizens in London. Ageing Soc 1:1-23
132. Grenier AM, Guberman N (2009) Creating and sustaining disadvantage: The relevance of a social exclusion framework. Health Soc Care Comm 17:116-124
133. Grewal I, Nazroo J, Bajekal M et al. (2004) Influences on quality of life: A qualitative investigation of ethnic differences among older people in England. J Ethn Migr Stud 30:737-761
134. Grundy E, Sloggett A (2003) Health inequalities in the older population: the role of personal capital, social resources and socio-economic circumstances. Soc Sci Med 56:935-947
135. Haan MN, Al-Hazzouri AZ, Aiello AE (2011) Life-span socioeconomic trajectory, nativity, and cognitive aging in Mexican Americans: The Sacramento Area Latino Study on Aging. J Gerontol B-Psychol 66:102-110
136. Habib KN (2014) An investigation on mode choice and travel distance demand of older people in the National Capital Region (NCR) of Canada: application of a utility theoretic joint econometric model. Transportation 42:143-161
137. Halley E, Mulligan E, Pratt E et al. (2010) Older Irish people with dementia in England. Advances in Mental Health 9:221-232
138. Hand C, Law M, Hanna S et al. (2012) Neighbourhood influences on participation in activities among older adults with chronic health conditions. Health Place 18:869-876
139. Harley DA, Gassaway L, Dunkley L (2016) Isolation, socialization, recreation, and inclusion of LGBT elders. In: Harley DA, Teaster PB (eds) Handbook of LGBT Elders: An interdisciplinary approach to principles, practices, and policies, Springer International Publishing Switzerland, pp 563-581
140. Harrison J (1999) Lavender pink grey power: gay and lesbian gerontology in Australia. Australas J Ageing 18:32-37
141. Haustein S, Siren A (2014) Seniors’ unmet mobility needs – how important is a driving licence? J Transp Geogr 41:45-52
142. Hawkley LC, Thisted RA, Cacioppo JT (2009) Loneliness predicts reduced physical activity: Cross-sectional & longitudinal analyses. Health Psychol 28:354-363
143. Hawkley LC, Thisted RA, Masi CM et al. (2010) Loneliness predicts increased blood pressure: 5-Year cross-lagged analyses in middle-aged and older adults. Psychol Aging 25:132-142
144. Heap J, Fors S (2015) Duration and accumulation of disadvantages in old age. Soc Indic Res 123:411-429
145. Heap J, Lennartsson C, Thorslund M (2013) Coexisting disadvantages across the adult age span: A comparison of older and younger age groups in the Swedish welfare state. Int J Soc Welf 22:130-140
146. Heenan D (2010) Social capital and older people in farming communities. J Aging Stud 24:40-46
147. Heikkinen SJ (2011) Exclusion of older immigrants from the former Soviet Union to Finland: The meaning of intergenerational relationships. Journal of Cross-Cultural Gerontology 26:379-395
148. Hendricks J (2004) Public policies and old age identity. J Aging Stud 18:245-260
149. Heslop A (1999) Ageing and development. Dept. for International Development, London
150. Hirshorn BA, Settersten Jr RA (2013) Civic involvement across the life course: Moving beyond age-based assumptions. Adv Life Course Res 18:199-211
151. Hlebec V, Mali J, Hrast MF (2014) Community care for older people in Slovenia. Anthropological Notebooks 20:5-20
152. Hoff A (2008) Tackling poverty and social exclusion of older people: Lessons from Europe: Oxford Institute of Ageing, Oxford
153. Hoi LV, Phuc HD, Dung TV et al. (2009) Remaining life expectancy among older people in a rural area of Vietnam: trends and socioeconomic inequalities during a period of multiple transitions. Bmc Public Health 9:471
154. Holstein MB (2001) Feminist perspective on anti-aging medicine. Generations 25:38-43
155. Hossen A, Westhues A (2010) A socially excluded space: Restrictions on access to health care for older women in rural Bangladesh. Qual Health Res 20:1192-1201
156. Hrast MF, Hlebec V, Kavcic M (2012) The social exclusion of the elderly: A mixed-methods study in Slovenia. Sociol Cas 48:1051-1074
157. Hrast MF, Mrak AK, Rakar T (2013) Social exclusion of elderly in Central and Eastern Europe. Int J Soc Econ 40:971-989
158. Hugman R (2001) Ageing in space. Australas J Ageing 20:57-65
159. Hui ECM, Wong FKW, Chung KW et al. (2014) Housing affordability, preferences and expectations of elderly with government intervention. Habitat Int 43:11-21
160. Hunkler C, Kneip T, Sand G, Schuth M (2015) Growing old abroad: social and material deprivation among first- and second generation migrants in Europe. In: Börsch-Supan A, Kneip T, Litwin H, Myck M, Weber G (eds) Ageing in Europe - supporting policies for an inclusive society. DE GRUYTER, pp 199-208
161. Inder KJ, Lewin TJ, Kelly BJ (2012) Factors impacting on the well-being of older residents in rural communities. Perspect Public Heal 132:182-191
162. Innes A, Cox S, Smith A et al. (2006) Service provision for people with dementia in rural Scotland Difficulties and innovations. Dementia 5:249-270
163. Irwin S (1999) Later life, inequality and sociological theory. Ageing Soc 19:691-715
164. Jatrana S, Blakely T (2014) Socio-economic inequalities in mortality persist into old age in New Zealand: study of all 65 years plus, 2001-04. Ageing Soc 34:911-929
165. Jiménez MP, Osypuk TL, Arevalo S et al. (2015) Neighborhood socioeconomic context and change in allostatic load among older Puerto Ricans: The Boston Puerto Rican health study. Health Place 33:1-8
166. Johnstone M-J, Kanitsaki O (2008) Ethnic aged discrimination and disparities in health and social care: A question of social justice. Australas J Ageing 27:110-115
167. Jokinen-Gordon H (2012) Still penalized? Parity, age at first birth and women's income in later life. J Women Aging 24:227-241
168. Jones A, Goodman A, Roberts H et al. Entitlement to concessionary public transport and wellbeing: A qualitative study of young people and older citizens in London, UK. Soc Sci Med 91:202-209
169. Jürges H (2015) Health insurance coverage and access to care among European elders: cross national differences and social gradients. In: Börsch-Supan A, Kneip T, Litwin H, Myck M, Weber G (eds) Ageing in Europe - supporting policies for an inclusive society. DE GRUYTER, pp 301-312
170. Kahn JR, Fazio EM (2005) Economic status over the life course and racial disparities in health. J Gerontol B-Psychol 60B:76-84
171. Kaplan MS, Huguet N, Feeny DH et al. (2010) Self-reported hypertension prevalence and income among older adults in Canada and the United States. Soc Sci Med 70:844-849
172. Keating N, Eales J, Phillips JE (2013) Age-friendly rural communities: Conceptualizing ‘Best-Fit’. Can J Aging 32:319-332
173. Keene DE, Ruel E (2013) “Everyone called me grandma”: Public housing demolition and relocation among older adults in Atlanta. Cities
174. Kemperman A, Timmermans H (2014) Green spaces in the direct living environment and social contacts of the aging population. Landscape Urban Plan 129:44-54
175. Kendig H, Quine S, Russell C et al. (2004) Health promotion for socially disadvantaged groups: the case of homeless older men in Australia. Health Promot Int 19:157-165
176. Kim J, Richardson V, Park B et al. (2013) A multilevel perspective on gender differences in the relationship between poverty status and depression among older adults in the United States. J Women Aging 25:207-226
177. Kim S (2011) Assessing mobility in an aging society: Personal and built environment factors associated with older people’s subjective transportation deficiency in the US. Transport Res F-Traf 14:422-429
178. King S, Dabelko-Schoeny H (2009) “Quite Frankly, I Have Doubts About Remaining”: Aging-in-place and health care access for rural midlife and older lesbian, gay, and bisexual individuals. Journal of LGBT Health Research 5:10-21
179. Klesges LM, Pahor M, Shorr RI et al. (2001) Financial difficulty in acquiring food among elderly disabled women: Results from the Women's Health and Aging Study. Am J Public Health 91:68-75
180. Kossioni AE (2012) Is Europe prepared to meet the oral health needs of older people? Gerodontology 29:E1230-E1240
181. Krause N (2006) Neighborhood Deterioration, social skills, and social relationships in late life. Int J Aging Hum Dev 62:185-207
182. Kreager P (2006) Migration, social structure and old-age support networks: a comparison of three Indonesian communities. Ageing Soc 26:37-60
183. Kröger H, Hoffmann R (2015) Who can realise their retirement plans? Poor health and employment crises as factors of exclusion. In: Börsch-Supan A, Kneip T, Litwin H, Myck M, Weber G (eds) Ageing in Europe - supporting policies for an inclusive society. DE GRUYTER, pp 115-126
184. Krout JA (1997) Barriers to providing case management to older rural persons. Journal of Case Management 6:142-150
185. Kubzansky LD, Subramanian S, Kawachi I et al. (2005) Neighborhood contextual influences on depressive symptoms in the elderly. Am J Epidemiol 162:253-260
186. Kwag KH, Jang Y, Rhew SH et al. (2011) Neighborhood effects on physical and mental health: A study of Korean American older adults. Asian American Journal of Psychology 2:91-100
187. Ladin K, Daniels N, Kawachi I (2010) Exploring the relationship between absolute and relative position and late-life depression: Evidence from 10 European countries. Gerontologist 50:48-59
188. Laferrère A, Bosch K (2015) Unmet need for long-term care and social exclusion. In: Börsch-Supan A, Kneip T, Litwin H, Myck M, Weber G (eds) Ageing in Europe - supporting policies for an inclusive society. DE GRUYTER, pp 331-342
189. Lager D, Van Hoven B, Huigen PPP (2013) Dealing with change in old age: Negotiating working-class belonging in a neighbourhood in the process of urban renewal in the Netherlands. Geoforum 50:54-61
190. Lager D, Van Hoven B, Huigen PPP (2015) Understanding older adults’ social capital in place: Obstacles to and opportunities for social contacts in the neighbourhood. Geoforum 59:87-97
191. Lai DWL (2011) Perceived impact of economic downturn on worry experienced by elderly Chinese immigrants in Canada. Journal of Family and Economic Issues 32:521-531
192. Laliberte RD (2015) Embodying positive aging and neoliberal rationality: Talking about the aging body within narratives of retirement. J Aging Stud 34:10-20
193. Lam LT, Lam MK (2003) Associations between social, economic resources and mental, physical illnesses in a population of older people in New South Wales, Australia. Journal of Mental Health and Aging 9:223-232
194. Lang IA, Gibbs SJ, Steel N et al. (2008) Neighbourhood deprivation and dental service use: a cross-sectional analysis of older people in England. J Public Health 30:472-478
195. Lee M-C, Huang N (2015) Changes in self-perceived economic satisfaction and mortality at old ages: Evidence from a survey of middle-aged and elderly adults in Taiwan. Soc Sci Med 130:1-8
196. Lee Y, Hong PYP, Harm Y (2014) Poverty among Korean immigrant older adults: Examining the effects of Social Exclusion. J Soc Serv Res 40:385-401
197. Lei X, Sun X, Strauss J et al. (2014) Health outcomes and socio-economic status among the mid-aged and elderly in China: Evidence from the CHARLS national baseline data. The Journal of the Economics of Ageing 3:29-43
198. Leone T, Hessel P (2015) The effect of social participation on the subjective and objective health status of the over-fifties: evidence from SHARE. Ageing Soc 36:968-987
199. Li Y and Robert SA (2008) The contributions of race, individual socioeconomic status, and neighborhood socioeconomic context on the self-rated health trajectories and mortality of older adults. Res Aging 30:251-273
200. Lissitsa S, Chachashvili-Bolotin S (2015) Does the wind of change blow in late adulthood? Adoption of ICT by senior citizens during the past decade. Poetics 52:44-63
201. Liu G-J, Engels B (2012) Accessibility to essential services and facilities by a spatially dispersed aging population in suburban Melbourne, Australia. In: Gartner G, Ortag F (eds) Advances in Location-Based Services. Springer Verlag, Berlin/Heidelberg, pp 327-348
202. Liu L, Newschaffer CJ (2011) Impact of social connections on risk of heart disease, cancer, and all-cause mortality among elderly Americans: Findings from the Second Longitudinal Study of Aging (LSOA II). Arch Gerontol Geriat 53:168-173
203. Liu L, Gou Z, Zuo J (2014) Social support mediates loneliness and depression in elderly people. J Health Psychol. doi: 10.1177/1359105314536941
204. Lloyd-Sherlock P, Barrientos A, Moller V et al. (2012) Pensions, poverty and wellbeing in later life: Comparative research from South Africa and Brazil. J Aging Stud 26:243-252
205. Lloyd-Sherlock P (2000) Old age and poverty in developing countries: New Policy Challenges. World Dev 28:2157-2168
206. Lloyd-Sherlock P (2002) Formal social protection for older people in developing countries: three different approaches. J Soc Policy 31:695-713
207. Lou VWQ (2010) Life satisfaction of older adults in Hong Kong: The role of social support from grandchildren. Soc Indic Res 95:377-391
208. Lui CW, Warburton J, Winterton R et al. (2011) Critical reflections on a social inclusion approach for an ageing Australia. Aust Soc Work 64:266-282
209. Luo Y, Waite LJ (2014) Loneliness and mortality among older adults in China. J Gerontol B-Psychol 69:633-645
210. Manthorpe J, Iliffe S, Clough R et al. (2008) Elderly people’s perspectives on health and well-being in rural communities in England: Findings from the evaluation of the national service framework for older people. Health Soc Care Comm 16:460-468
211. Manthorpe J, Iliffe S, Moriarty J et al. (2009) ‘We are not blaming anyone, but if we don't know about amenities, we cannot seek them out’: black and minority older people's views on the quality of local health and personal social services in England. Ageing Soc 29:93-113
212. Marquet O, Miralles-Guasch C (2015) Neighbourhood vitality and physical activity among the elderly: The role of walkable environments on active ageing in Barcelona, Spain. Soc Sci Med 135:24-30
213. McCann E, Sharek D, Higgins A et al. (2013) Lesbian, gay, bisexual and transgender older people in Ireland: Mental health issues. Aging Ment Health 17:358-365
214. McDonald A, Heath B (2008) Developing services for people with dementia: Findings from research in a rural area. Quality in Ageing - Policy, practice and research 9:9-18
215. McDonald S, Mair CA (2010) Social capital across the life course: age and gendered patterns of network resources. Sociol Forum 25:335-359
216. McDonough P, Sacker A, Wiggins RD (2005) Time on my side? Life course trajectories of poverty and health. Soc Sci Med 61:1795-1808
217. McLaughlin D, Vagenas D, Pachana NA et al. (2010) Gender differences in social network size and satisfaction in adults in their 70s. J Health Psychol 15:671-679
218. McNamara JM (2007) Long-term disadvantage among elderly women: The effects of work history. Soc Serv Rev 81:423-452
219. McVittie C, McKinlay A, Widdicombe S (2008) Passive and active non-employment: Age, employment and the identities of older non-working people. J Aging Stud 22:248-255
220. Meinow B, Parker MG, Thorslund M (2011) Consumers of eldercare in Sweden: The semblance of choice. Soc Sci Med 73:1285-1289
221. Menec VH, Veselyuk DM, Blandford AA et al. (2009) Availability of activity-related resources in senior apartments: does it differ by neighbourhood socio-economic status? Ageing Soc 29:397-411
222. Meyer T, Bridgen P (2008) Class, gender and chance: the social division of welfare and occupational pensions in the United Kingdom. Ageing Soc 28:353-381
223. Milbourne P, Doheny S (2012) Older people and poverty in rural Britain: Material hardships, cultural denials and social inclusions. J Rural Stud 28:389-397
224. Milne A, Williams J (2000) Meeting the mental health needs of older women: taking social inequality into account. Ageing Soc 20:699-723
225. Milne A, Hatzidimitriadou E, Wiseman J (2007) Health and quality of life among older people in rural England: Exploring the impact and efficacy of policy. J Soc Policy 36:477-495
226. Milner PC, Payne JN, Stanfield RC et al. (2004) Inequalities in accessing hip joint replacement for people in need. Eur J Public Health 14:58-62
227. Moffatt S, Glasgow N (2009) How useful is the concept of social exclusion when applied to rural older people in the United Kingdom and the United States? Reg Stud 43:1291-1303
228. Moffatt S, Mackintosh J (2009) Older people's experience of proactive welfare rights advice: Qualitative study of a South Asian community. Ethnic Health 14:5-25
229. Moffatt S, Scambler G (2008) Can welfare-rights advice targeted at older people reduce social exclusion? Ageing Soc 28:875-899
230. Morris A (2009) Living on the margins: Comparing older private renters and older public housing tenants in Sydney, Australia. Housing Stud 24:693-707
231. Morris A (2012) Older social and private renters, the neighbourhood, and social connections and activity. Urban Policy and Research 30:43-58
232. Mulley G (2007) Myths of ageing. Clin Med 7:68-72
233. Najsztub M, Bonfatti A, Duda D (2015) Material and social deprivation in the macroeconomic context. In: Börsch-Supan A, Kneip T, Litwin H, Myck M, Weber G (eds) Ageing in Europe - supporting policies for an inclusive society. DE GRUYTER, pp 79-90
234. Naz S, Gul S (2014) Relationship between economic independence, social support and quality of life among elderly people. Journal of the Indian Academy of Applied Psychology 40:255-260
235. Needham BL, Carroll JE, Diez Roux AV et al. (2014) Neighborhood characteristics and leukocyte telomere length: The Multi-Ethnic Study of Atherosclerosis. Health Place 28:167-172
236. Newall NEG, Chipperfield JG, Bailis DS et al. (2013) Consequences of loneliness on physical activity and mortality in older adults and the power of positive emotions. Health Psychol 32:921-924
237. Ní Léime Á, Connolly S (2015) Active ageing: social participation and volunteering in later life. In: Walsh K, Carney G, Ní Léime Á (eds) Ageing through austerity: critical perspectives from Ireland. Policy Press, Bristol, pp 47-62
238. Ní Léime Á, Duvvury N, Callan A (2015) Pension provision, gender, ageing and work in Ireland. In: Walsh K, Carney G, Ní Léime Á (eds) Ageing through austerity: Critical perspectives from Ireland. Policy Press, Bristol, pp 63-78
239. Nordbakke S, Schwanen T (2015) Transport, unmet activity needs and wellbeing in later life: exploring the links. Transportation 42:1129-1151
240. North MS, Fiske ST (2013) Subtyping ageism: Policy issues in succession and consumption. Social Issues and Policy Review 7:36-57
241. O’Shea E, Cahill S, Pierce M (2015) Reframing policy for dementia. In: Walsh K, Carney G, Ní Léime Á (eds) Ageing through austerity: Critical perspectives from Ireland. Policy Press, Bristol, pp 97-112
242. Ogg J, Renaut S (2012) Social inclusion of elders in families. In: Scharf T, Keating N (eds) From exclusion to inclusion in old age: A global challenge. Policy Press, Bristol, pp 89-108
243. Ogg J (2003) Living alone in later life. Institute of Community Studies, London
244. Oh JH, Kim S (2009) Aging, neighborhood attachment, and fear of crime: Testing reciprocal effects. J Community Psychol 37:21-40
245. Olphert W, Damodaran L (2013) Older people and digital disengagement: a fourth digital divide? Gerontology 59:564-570
246. O'Neill T, Jinks C, Squire A (2006) "Heating is more important than food": Older women's perceptions of fuel poverty. Journal of Housing for the Elderly 20:95-108
247. O’Rand AM (2006) Nine - Stratification and the life course: Life course capital, life course risks, and social inequality. In: Robert HB, Linda KG, Stephen JC et al (eds) Handbook of aging and the social sciences (Sixth Edition). Academic Press, Burlington, pp 145-162
248. O’Reilly D (2002) Standard indicators of deprivation: do they disadvantage older people? Age Ageing 31:197-202
249. O’Shea E, Walsh K, Scharf T (2012) Exploring community perceptions of the relationship between age and social exclusion in rural areas. Quality in Ageing and Older Adults 13:16-26
250. Österholm JH, Samuelsson C (2015) Orally positioning persons with dementia in assessment meetings. Ageing Soc 35:367-388
251. Oswald F, Jopp D, Rott C et al. (2011) Is aging in place a resource for or risk to life satisfaction? Gerontologist 51:238-250
252. Otis MD, Harley DA (2016) The intersection of identities of lgbt elders: Race, age, sexuality, and care network. In: Harley DA, Teaster PB (eds) Handbook of LGBT Elders: An interdisciplinary approach to principles, practices, and policies. Springer International Publishing Switzerland, pp 83-101
253. Özmete E (2015) Measuring the poverty of elderly people with needs analysis in Turkey. In: Copur Z (ed) Handbook of research on behavioral finance and investment strategies: Decision making in the inancial industry. Business Science Reference, Hershey PA, pp 127-146
254. Paez A, Mercado RG, Farber S et al. (2010) Accessibility to health care facilities in Montreal Island: an application of relative accessibility indicators from the perspective of senior and non-senior residents. Int J Health Geogr 9:52
255. Pandey MK (2012) Poverty and disability among Indian elderly: Evidence from household survey. Journal of Disability Policy Studies 23:39-49
256. Paoletti I, de Carvalho MI (2012) Ageing, poverty and social services in Portugal: The importance of quality services. Indian Journal of Gerontology 26:396-413
257. Park NS, Jang Y, Lee BS et al. (2014) The impact of social resources on depressive symptoms in racially and ethnically diverse older adults: Variations by groups with differing health risks. Res Aging 36:322-342
258. Parmar D, Williams G, Dkhimi F et al. (2014) Enrolment of older people in social health protection programs in West Africa - Does social exclusion play a part? Soc Sci Med 119:36-44
259. Patel KV, Eschbach K, Rudkin LL et al. (2003) Neighborhood context and self-rated health in older Mexican Americans. Ann Epidemiol 13:620-628
260. Patsios D, Hillyard P, Machniewski S et al. (2012) Inequalities in old age: the impact of the recession on older people in Ireland, North and South. Quality in ageing and older adults 13:27-37
261. Patsios D (2014) Trends in older people's perceptions of necessities and deprivation in Great Britain and Northern Ireland: What difference did a decade (or so) make? Journal of Poverty and Social Justice 22:227-251
262. Patulny R (2009) The golden years? Social isolation among retired men and women in Australia. Family Matters 83:39-47
263. Peace S, Holland C (2001) Housing an ageing society. In: Peace S, Holland C (eds) Inclusive housing in an ageing society. Policy Press, Bristol, pp 1-26
264. Peeters H, Debels A and Verpoorten R. (2013) Excluding institutionalized elderly from surveys: Consequences for income and poverty statistics. Soc Indic Res 110:751-769
265. Percival J, Hanson J (2005) 'I'm like a tree a million miles from the water's edge': Social care and inclusion of older people with visual impairment. Brit J Soc Work 35:189-205
266. Petriwskyj A, Warburton J, Everingham J-A et al. (2012) Diversity and inclusion in local governance: An Australian study of seniors' participation. J Aging Stud 26:182-191
267. Phelan A (2008) Elder abuse, ageism, human rights and citizenship: implications for nursing discourse. Nurs Inq 15:320-329
268. Phillips J, Marks G. (2008) Ageing lesbians: Marginalising discourses and social exclusion in the aged care industry. Journal of Gay and Lesbian Social Services 20:187-202
269. Phillipson C, Allan GA, Morgan DHJ (2004) Social networks and social exclusion: sociological and policy perspectives. Ashgate Publishing Company
270. Phillipson C (2007) The ‘elected’ and the ‘excluded’: sociological perspectives on the experience of place and community in old age. Ageing Soc 27:321-342
271. Pizzetti P, Manfredini M (2008) "The shock of widowhood"? Evidence from an Italian population (Parma, 1989-2000). Soc Indic Res 85:499-513
272. Porter G, Tewodros A, Bifandimu F et al. (2013) Transport and mobility constraints in an aging population: health and livelihood implications in rural Tanzania. J Transp Geogr 30:161-169
273. Postle K, Beresford P (2007) Capacity building and the reconception of political participation: A role for social care workers? Brit J Soc Work 37:143-158
274. Postle K, Wright P, Beresford P (2005) Older people's participation in political activity--Making their voices heard: A potential support role for welfare professionals in countering ageism and social exclusion. Practice 17:173-189
275. Prada SI, Duarte JL, Guerrero R (2015) Out-of-pocket health expenditure for poor and non-poor older adults in Colombia: Composition and trends. International Journal of Consumer Studies 39:362-368
276. Price D (2006) The poverty of older people in the UK. J Soc Work Pract 20:251-266
277. Prince MJ, Harwood RH, Blizard RA et al (1997) Social support deficits, loneliness and life events as risk factors for depression in old age. The Gospel Oak Object. Psychol Med 27:323-332
278. Prokos AH, Keene JR (2012) The life course and cumulative disadvantage: Poverty among grandmother-headed families. Res Aging 34:592-621
279. Pruchno RA, Wilson-Genderson M, Cartwright FP (2012) The texture of neighborhoods and disability among older adults. J Gerontol B-Psychol 67:89-98
280. Qin N, Yan E (2014) Prevalence and psychosocial correlates of the fear of crime in older Chinese. Journal of Adult Protection 16:264-275
281. Ranzijn R (2010) Active ageing-Another way to oppress marginalized and disadvantaged elders? Aboriginal elders as a case study. J Health Psychol 15:716-723
282. Ravulaparthy S, Yoon S, Goulias K (2013) Linking elderly transport mobility and subjective well-being. Transp Res Record 2382:28-36
283. Raymond E, Grenier A (2013) Participation in policy discourse: New form of exclusion for seniors with disabilities? Can J Aging 32:117-129
284. Riach K, Loretto W (2009) Identity work and the 'unemployed' worker: Age, disability and the lived experience of the older unemployed. Work Employ Soc 23:102-119
285. Rogers M, Winterton R, Warburton J et al. (2015) Water management and healthy ageing in rural Australia economic, social, and cultural considerations. Environ Behav 47:551-569
286. Roh S, Jang Y, Chiriboga DA et al. (2011) Perceived neighborhood environment affecting physical and mental health: A study with Korean American older adults in New York City. Journal of Immigrant and Minority Health 13:1005-1012
287. Rozanova J, Keating N, Eales J (2012) Unequal social engagement for older adults: Constraints on choice. Can J Aging 31:25-36
288. Rozanova J (2010) Discourse of successful aging in The Globe & Mail: Insights from critical gerontology. J Aging Stud 24:213-222
289. Russell C, Porter M (2003) Single older men in disadvantaged households: narratives of meaning around everyday life. Ageing International 28:359-371
290. Russell C, Hill B, Basser M (1998) Older people's lives in the inner city: hazardous or rewarding? Aust NZ J Publ Heal 22:98-106
291. Ryser L, Halseth G (2011) Informal support networks of low-income senior women living alone: Evidence from Fort St. John, BC. J Women Aging 23:185-202
292. Ryser L, Halseth G (2012) Resolving mobility constraints impeding rural seniors' access to regionalized services. Journal of Aging & Social Policy 24:328-344
293. Ryvicker M, Gallo WT, Fahs MC (2012) Environmental factors associated with primary care access among urban older adults. Soc Sci Med 75:914-921
294. Sabik NJ (2015) Ageism and body esteem: Associations with psychological well-being among late middle-aged African American and European American Women. J Gerontol B-Psychol 70:189-199
295. Saito M, Kondo N, Kondo K et al. (2012) Gender differences on the impacts of social exclusion on mortality among older Japanese: AGES cohort study. Soc Sci Med 75:940-945
296. Samuel LJ, Glass TA, Thorpe Jr RJ et al. (2015) Household and neighborhood conditions partially account for associations between education and physical capacity in the National Health and Aging Trends Study. Soc Sci Med 128:67-75
297. Sandoval DA, Rank MR, Hirschl TA (2009) The increasing risk of poverty across the American life course. Demography 46:717-737
298. Sarabia-Cobo CM, Castanedo Pfeiffer C (2015) Changing negative stereotypes regarding aging in undergraduate nursing students. Nurs Educ Today 35:e60-e64
299. Saunders P, Lujun S (2006) Poverty and hardship among the aged in urban China. Soc Policy Admin 40:138-157
300. Scarborough BK, Like-Haislip TZ, Novak KJ et al. (2010) Assessing the relationship between individual characteristics, neighborhood context, and fear of crime. J Crim Just 38:819-826
301. Scharf T, DeJ ong Gierveld J (2008) Loneliness in urban neighbourhoods: An Anglo-Dutch comparison. Eur J Ageing 5:103-115
302. Scharf T, Bartlam B (2008) Ageing and social exclusion in rural communities. In: Keating N (ed) Rural ageing: a good place to grow old? Policy Press, Bristol, pp 97-108
303. Scharf T, Phillipson C, Smith A (2003) Older people's perceptions of the neighbourhood: Evidence from socially deprived urban areas. Sociological Research Online 8
304. Scharf T, Phillipson C, Smith AE (2004) Poverty and social exclusion: Growing older in deprived urban neighbourhoods. In: Walker A, Hagan Hennessy C (eds) Growing older: Quality of life in old age. Open University Press, pp 81-106
305. Scharf T, Phillipson C, Smith AE (2005) Social exclusion of older people in deprived urban communities of England. Eur J Ageing 2:76-87
306. Scharf T, Phillipson C, Kingston P et al. (2001) Social exclusion and older people: exploring the connections. Education and Ageing 16:303-320
307. Scharf T, Phillipson C, Smith A, Kingston, P (2002) Growing older in socially deprived areas: Social exclusion in later life. Helped the Aged
308. Scharf T. (2005) Multiple exclusion and quality of life amongst excluded older people in disadvantaged neighbourhoods. Office of the Deputy Prime Minister
309. Scharlach AE, Lehning AJ (2013) Ageing-friendly communities and social inclusion in the United States of America. Ageing Soc 33:110-136
310. Schieman S, Pearlin LI (2006) Neighborhood disadvantage, social comparisons, and the subjective assessment of ambient problems among older adults. Soc Psychol Quart 69:253-269
311. Schieman S (2009) Residential stability, neighborhood racial composition, and the subjective assessment of neighborhood problems among older adults. Sociol Quart 50:608-632
312. Schoellgen I, Huxhold O, Schuez B et al. (2011) Resources for health: Differential effects of optimistic self-beliefs and social support according to socioeconomic status. Health Psychol 30:326-335
313. Schwanen T, Banister D, Bowling A (2012) Independence and mobility in later life. Geoforum 43:1313-1322
314. Scourfield P (2007) Helping older people in residential care remain full citizens. Brit J Soc Work 37:1135-1152
315. Serrat R, Villar F, Celdrán M (2015) Factors associated with Spanish older people’s membership in political organizations: the role of active aging activities. Eur J Ageing 12:239-247
316. Shankar A, Hamer M, McMunn A et al. (2013) Social isolation and loneliness: Relationships with cognitive function during 4 years of follow-up in the English longitudinal study of ageing. Psychosomatic Medicine 75:161-170
317. Shankardass MK (2003) Concern for ageing women in India. Bold 13:19-24
318. Sharek DB, McCann E, Sheerin F et al. (2015) Older LGBT people's experiences and concerns with healthcare professionals and services in Ireland. International Journal of Older People Nursing 10:230-240
319. Shaw BA, McGeever K, Vasquez E et al. (2014) Socioeconomic inequalities in health after age 50: Are health risk behaviors to blame? Soc Sci Med 101:52-60
320. Shen K, Zeng Y (2014) Direct and indirect effects of childhood conditions on survival and health among male and female elderly in China. Soc Sci Med 119:207-214
321. Shergold I, Parkhurst G (2010) Operationalising 'sustainable mobility': the case of transport policy for older citizens in rural areas. J Transp Geogr 18:336-339
322. Shergold I, Parkhurst G (2012) Transport-related social exclusion amongst older people in rural Southwest England and Wales. J Rural Stud 28:412-421
323. Shergold I, Parkhurst G, Musselwhite C (2012) Rural car dependence: An emerging barrier to community activity for older people. Transport Plan Techn 35:69-85
324. Shiovitz-Ezra S (2015) Loneliness in Europe: do perceived neighbourhood characteristics matter? In: Börsch-Supan A, Kneip T, Litwin H, Myck M, Weber G (eds) Ageing in Europe - supporting policies for an inclusive society. DE GRUYTER, pp 169-178
325. Shirahase S (2015) Income inequality among older people in rapidly aging Japan. Research in Social Stratification and Mobility 41:1-10
326. Simms M (2004) A theory of age exclusion through closure: ‘Chronological age’ to ‘clinical need’. J Aging Stud 18:445-465
327. Smith AE, Sim J, Scharf T et al. (2004) Determinants of quality of life amongst older people in deprived neighbourhoods. Ageing Soc 24:793-814
328. Smith AE (2009) Ageing in urban neighbourhoods: place attachment and social exclusion. Policy Press
329. Smith JP, Tian M, Zhao Y (2013) Community effects on elderly health: Evidence from CHARLS national baseline. The Journal of the Economics of Ageing 1–2:50-59
330. Solway E, Estes CL, Goldberg S et al. (2010) Access barriers to mental health services for older adults from diverse populations: Perspectives of leaders in mental health and aging. Journal of Aging & Social Policy 22:360-378
331. Son J, Yarnal C, Kerstetter D (2010) Engendering social capital through a leisure club for middle-aged and older women: Implications for individual and community health and well-being. Leisure Studies 29:67-83
332. Srakar A, Hrast MF, Hlebec V, Majcen B (2015) Social exclusion, welfare regime and unmet long-term care need: evidence from SHARE. In: Börsch-Supan A, Kneip T, Litwin H, Myck M, Weber G (eds) Ageing in Europe - supporting policies for an inclusive society. DE GRUYTER, pp 189-198
333. Stafford M, McMunn A, De Vogli R (2011) Neighbourhood social environment and depressive symptoms in mid-life and beyond. Ageing Soc 31:893-910
334. Stephens C, Alpass F, Towers A (2010) Economic hardship among older people in New Zealand: The effects of low living standards on social support, loneliness, and mental health. New Zeal J Psychol 39:49-55
335. Stephens C, Breheny M, Mansvelt J (2015) Healthy ageing from the perspective of older people: A capability approach to resilience. Psychol Health 30:715-731
336. Stephens C, Breheny M, Mansvelt J (2015a) Volunteering as reciprocity: Beneficial and harmful effects of social policies to encourage contribution in older age. J Aging Stud 33:22-27
337. Stjernborg V, Wretstrand A, Tesfahuney M (2015) Everyday life mobilities of older Persons – A case study of ageing in a suburban landscape in Sweden. Mobilities 10:383-401
338. Stoeckel KJ, Litwin H (2015) Accessibility to neighbourhood services and well-being among older Europeans. In: Börsch-Supan A, Kneip T, Litwin H, Myck M, Weber G (eds) Ageing in Europe - supporting policies for an inclusive society. DE GRUYTER, pp 39-48
339. Stoeckel KJ, Litwin H (2015) Social cohesiveness and neighbourhood environmental deprivation: how are they related to life satisfaction in late life? In: Börsch-Supan A, Kneip T, Litwin H, Myck M, Weber G (eds) Ageing in Europe - supporting policies for an inclusive society. DE GRUYTER, pp 149-158
340. Street D (1997) Special interests or citizens' rights? ''Senior power,'' social security, and Medicare. Int J Health Serv 27:727-751
341. Sugiyama T, Ward Thompson C (2007) Older people's health, outdoor activity and supportiveness of neighbourhood environments. Landscape Urban Plan 83:168-175
342. Sugiyama T, Ward Thompson C (2008) Associations between characteristics of neighbourhood open space and older people's walking. Urban For Urban Gree 7:41-51
343. Suurmond J, Rosenmöller DL, el Mesbahi H et al. (2015) Barriers in access to home care services among ethnic minority and Dutch elderly – A qualitative study. Int J Nurs Stud 54:23-35
344. Tai TO, Treas J (2009) Does household composition explain welfare regime poverty risks for older adults and other household members? J Gerontol B-Psychol 64:777-787
345. Tam S, Neysmith S (2006) Disrespect and isolation: elder abuse in Chinese communities. Can J Aging 25:141-152
346. Tanner D (2003) Older people and access to care. Brit J Soc Work 33:499-515
347. Taylor MA, Geldhauser HA (2007) Low-income older workers. In: Shultz KS, Adams GA (eds) Aging and work in 21^st^ century. Lawrence Erlbaum Associates, Mahway, NJ, pp 25-49
348. Taylor P, Walker A (1998) Employers and older workers: attitudes and employment practices. Ageing Soc 18:641-658
349. Tchernina NV, Tchernin EA (2002) Older people in Russia's transitional society: multiple deprivation and coping responses. Ageing Soc 22:543-562
350. Temelová J, Slezáková A (2014) The changing environment and neighbourhood satisfaction in socialist high-rise panel housing estates: The time-comparative perceptions of elderly residents in Prague. Cities 37:82-91
351. Thang LL (2015) Social networks and the wellbeing of older adults in Singapore. In: Cheng S-T, Chi I, Fung HH, Li LW, Woo J (eds) Successful Aging: Asian Perspectives, Springer Netherlands, pp 147-163
352. Tobiasz-Adamczyk B, Zawisza K (2015) Regional differences and determinants of social capital in Polish elders. Stud Socjol 2:119-141
353. Tomaszewski W (2013) Living environment, social participation and wellbeing in older age: the relevance of housing and local area disadvantage. Journal of Population Ageing 6:119-156
354. Tong H, Lai D, Zeng Q et al. (2011) Effects of social exclusion on depressive symptoms: elderly Chinese living alone in Shanghai, China. Journal of Cross-Cultural Gerontology 26:349-364
355. Treacy P, Butler M, Byrne A et al. (2004) Loneliness and social isolation among older Irish people. National Council on Ageing and Older People, Dublin
356. Treas J, Mazumdar S (2002) Older people in America's immigrant families: Dilemmas of dependence, integration, and isolation. J Aging Stud 16:243-258
357. Tur-Sinai A, Litwin H, Weber G (2015) Forgone visits to the doctor due to cost or lengthy waiting time among older adults in Europe. In: Börsch-Supan A, Kneip T, Litwin H, Myck M, Weber G (eds) Ageing in Europe - supporting policies for an inclusive society. DE GRUYTER, pp 291-300
358. Twigg J (2007) Clothing, age and the body: a critical review. Ageing Soc 27:285-306
359. Van Der Meer M, Fortuijn JD, Thissen F (2008) Vulnerability and environmental stress of older adults in deprived neighbourhoods in the Netherlands. Tijdschrift voor Economische en Sociale Geografie 99:53-64
360. van der Pas S, Ramklass S, O’Leary B et al. (2015) Features of home and neighbourhood and the liveability of older South Africans. Eur J Ageing 12:215-227
361. Van Dijk HM, Cramm JM, Nieboer AP (2014) Social cohesion as perceived by community-dwelling older people: the role of individual and neighbourhood characteristics. International Journal of Ageing and Later Life 8:9-31
362. van Dyk S (2014) The appraisal of difference: Critical gerontology and the active-ageing-paradigm. J Aging Stud 31:93-103
363. Van Sluytman LG, Torres D (2014) Hidden or uninvited? A content analysis of elder LGBT of color literature in gerontology. J Gerontol Soc Work 57:130-160
364. van Soest A, Zaidi A (2015) Old age work participation. In: Wright JD (ed) International Encyclopedia of the Social & Behavioral Sciences (Second Edition). Oxford: Elsevier, pp 182-189
365. Vecchio N (2008) The use of dental services among older Australians: Does location matter? Aust Econ Rev 41:272-282
366. Vera-Sanso P (2012) Gender, poverty and old-age livelihoods in urban South India in an era of globalisation. Oxford Development Studies 40:324-340
367. Victor CR, Bowling A (2012) A longitudinal analysis of loneliness among older people in Great Britain. J Psychol: Interdisciplinary and Applied 146:313-331
368. Victor CR, Burholt V, Martin W (2012) Loneliness and ethnic minority elders in Great Britain: An exploratory study. Journal of Cross-Cultural Gerontology 27:65-78
369. Victor CR, Scambler S, Bowling A et al. (2005) The prevalence of, and risk factors for, loneliness in later life: a survey of older people in Great Britain. Ageing Soc 25:357-375
370. Vignoli D, de Santis G (2010) Individual and contextual correlates of economic difficulties in old age in Europe. Popul Res Policy Rev 29:481-501
371. Vine D, Buys L, Aird R (2012) The use of amenities in high density neighbourhoods by older urban Australian residents. Landscape Urban Plan 107:159-171
372. Vitman A, Iecovich E, Alfasi N (2014) Ageism and social integration of older adults in their neighborhoods in Israel. Gerontologist 54:177-189
373. Vrooman GJGC (2008) Social exclusion of the elderly: A comparative study of EU Member States. Ceps
374. Wagner M, Brandt M (2015) Loneliness among informal caregivers aged 50+ in Europe. In: Börsch-Supan A, Kneip T, Litwin H, Myck M, Weber G (eds) Ageing in Europe - supporting policies for an inclusive society. DE GRUYTER, pp 179-188
375. Walker A, Walker C (1998) Normalisation and 'normal' ageing: the social construction of dependency among older people with learning difficulties. Disabil Soc 13:125-142
376. Walker J, Orpin P, Baynes H et al. (2013) Insights and principles for supporting social engagement in rural older people. Ageing Soc 33:938-963
377. Walker RB, Hiller JE (2007) Places and health: A qualitative study to explore how older women living alone perceive the social and physical dimensions of their neighbourhoods. Soc Sci Med 65:1154-1165
378. Walsh K (2015) Interrogating the ‘age-friendly community’ in austerity: myths, realties and the influence of place context. In: Walsh K, Carney G, Ní Léime Á (eds) Ageing through austerity: Critical perspectives from Ireland. Policy Press, Bristol, pp 79-95
379. Walsh K, Gannon B (2011) Perceived neighbourhood context, disability onset and old age. Journal of Socio-Economics 40:631-636
380. Walsh K, Shutes I (2013) Care relationships, quality of care and migrant workers caring for older people. Ageing Soc 33:393-420
381. Walsh K, Carney G, Ní Léime A (2015) Introduction – Social policy and ageing through austerity. In: Walsh K, Carney G, Ní Léime Á (eds) Ageing through austerity: Critical perspectives from Ireland. Policy Press, Bristol, pp 1-15
382. Walsh K, O’Shea E and Scharf T (2012a) Social exclusion and ageing in diverse rural communities: Findings from a cross-border study in Ireland and Northern Ireland: Irish Centre for Social Gerontology
383. Walsh K, O’Shea E, Scharf T, Murray M (2012b) Ageing in changing community contexts: Cross-border perspectives from rural Ireland and Northern Ireland. J Rural Stud 28:347-357
384. Walsh K, O'Shea E, Scharf T et al. (2011) Older people in rural communities: exploring attachment, contribution and diversity in rural Ireland and Northern Ireland. Ageing in Rural Communities (HARC) Research Network
385. Walsh K, O’Shea E, Scharf T, Shucksmith M (2014) Exploring the impact of informal practices on social exclusion and age-friendliness for older people in rural communities. J Community Appl Soc 24:37-49
386. Walter Rasugu Omariba D (2010) Neighbourhood characteristics, individual attributes and self-rated health among older Canadians. Health Place 16:986-995
387. Walters P, Bartlett H (2009) Growing old in a new estate: Establishing new social networks in retirement. Ageing Soc 29:217-236
388. Wanless D, Mitchell BA, Wister AV (2010) Social determinants of health for older women in Canada: Does rural-urban residency matter? Can J Aging 29:233-247
389. Warburton J, Cowan S, Winterton R et al. (2014) Building social inclusion for rural older people using information and communication technologies: Perspectives of rural practitioners. Aust Soc Work 67:479-494
390. Ward MR, Somerville P, Bosworth G (2013) 'Now without my car I don't know what I'd do': The transportation needs of older people in rural Lincolnshire. Local Economy 28:553-566
391. Warnes AM, Crane M (2006) The causes of homelessness among older people in England. Housing Stud 21:401-421
392. Waters J, Neale R (2010) Older people's perceptions of personal safety in deprived communities: understanding the social causes of fear of crime. Quality in Ageing and Older Adults 11:48-56
393. Waters WF, Gallegos CA (2014) Aging, health, and identity in Ecuador’s Indigenous communities. Journal of Cross-Cultural Gerontology 29:371-387
394. Weicht B (2013) The making of ‘the elderly’: Constructing the subject of care. J Aging Stud 27:188-197
395. Wen M, Cagney KA, Christakis NA (2005) Effect of specific aspects of community social environment on the mortality of individuals diagnosed with serious illness. Soc Sci Med 61:1119-1134
396. Wen M, Hawkley LC, Cacioppo JT (2006) Objective and perceived neighborhood environment, individual SES and psychosocial factors, and self-rated health: An analysis of older adults in Cook County, Illinois. Soc Sci Med 63:2575-2590
397. Wight RG, Aneshensel CS, Miller-Martinez D et al. (2006) Urban neighborhood context, educational attainment, and cognitive function among older adults. Am J Epidemiol 163:1071-1078
398. Wight RG, Cummings JR, Karlamangla AS et al. (2009) Urban neighborhood context and change in depressive symptoms in late life. J Gerontol B-Psychol 64:247-251
399. Wight RG, Cummings JR, Miller-Martinez D et al. (2008) A multilevel analysis of urban neighborhood socioeconomic disadvantage and health in late life. Soc Sci Med 66:862-872
400. Wight RG, Ko MJ, Aneshensel CS. (2011) Urban neighborhoods and depressive symptoms in late middle age. Res Aging 33:28-50
401. Wilińska M, Cedersund E (2010) “Classic ageism” or “brutal economy”? Old age and older people in the Polish media. J Aging Stud 24:335-343
402. Wilińska M, Henning C (2011) Old age identity in social welfare practice. Qualitative Social Work 10:346-363
403. Williams BO (2000) Ageism helps to ration medical treatment. Health bulletin 58:198-202
404. Wilson-Genderson M, Pruchno R (2013) Effects of neighborhood violence and perceptions of neighborhood safety on depressive symptoms of older adults. Soc Sci Med 85:43-49
405. Windle GS, Burholt V, Edwards RT (2006) Housing related difficulties, housing tenure and variations in health status: evidence from older people in Wales. Health Place 12:267-278
406. Winterton R, Clune S, Warburton J et al. (2014) Local governance responses to social inclusion for older rural Victorians: Building resources, opportunities and capabilities. Australas J Ageing 33:E8-E12
407. Woolham J, Daly G, Hughes E (2013) Loneliness amongst older people: findings from a survey in Coventry, UK. Quality in Ageing and Older Adults 14:192-204
408. Wray S (2003) Women growing older: Agency, ethnicity and culture. Sociology-the Journal of the British Sociological Association 37:511-527
409. Yan T, Escarce JJ, Liang L-J et al (2013) Exploring psychosocial pathways between neighbourhood characteristics and stroke in older adults: the cardiovascular health study. Age Ageing 42:391-397
410. Yao L, Robert SA (2008) The contributions of race, individual socioeconomic status, and neighborhood socioeconomic context on the self-rated health trajectories and mortality of older adults. Res Aging 30:251-273
411. Ye M, Chen Y (2014) The influence of domestic living arrangement and neighborhood identity on mental health among urban Chinese elders. Aging Ment Health 18:40-50
412. Ylli A (2010) Health and social conditions of older people in Albania: Baseline data from a national survey. Public Health Rev 32:549-560
413. Young AF, Russell A, Powers JR (2004) The sense of belonging to a neighbourhood: can it be measured and is it related to health and well being in older women? Soc Sci Med 59:2627-2637
414. Youn-Min P (2008) The missing gap between internet use and benefits: seniors limited internet experiences and social marginalization Development and Society 37:97-115
415. Yuan R and Ngai SS (2012) Social exclusion and neighborhood support: A case study of empty-nest elderly in urban Shanghai. J Gerontol Soc Work 55:587-608
416. Yunong H (2012) Family relations and life satisfaction of older people: A comparative study between two different hukous in China. Ageing Soc 32:19-40
417. Zaidi A (2008) Well-being of older people in ageing societies: Ashgate Publishing Company
418. Zajicek AM, Calasanti TM, Zajicek EK (2007) Pension reforms and old people in Poland: An age, class, and gender lens. J Aging Stud 21:55-68
419. Zebhauser A, Baumert J, Emeny RT et al. (2015) What prevents old people living alone from feeling lonely? Findings from the KORA-Age-study. Aging Ment Health 19:773-780
420. Zeitler E, Buys L (2015) Mobility and out-of-home activities of older people living in suburban environments: 'Because I'm a driver, I don't have a problem'. Ageing Soc 35:785-808
421. Zettel-Watson L, Britton M (2008) The impact of obesity on the social participation of older adults. J Gen Psychol 135:409-423
422. Zhang Z, Zhang J (2015) Social participation and subjective well-being among retirees in China. Soc Indic Res 123:143-160
423. Ziegler F (2012) “You have to engage with life, or life will go away”: An intersectional life course analysis of older women's social participation in a disadvantaged urban area. Geoforum 43:1296-1305
424. Zubair M, Norris M (2015) Perspectives on ageing, later life and ethnicity: Ageing research in ethnic minority contexts. Ageing Soc 35:897-91
425. Zunzunegui MV, Koné A, Johri M et al. (2004) Social networks and self-rated health in two French-speaking Canadian community dwelling populations over 65. Soc Sci Med 58:2069-2081
